# Kinesin‐Driven De‐Mixing of Cytoskeleton Composites Drives Emergent Mechanical Properties

**DOI:** 10.1002/marc.202401128

**Published:** 2025-04-10

**Authors:** Janet Sheung, Christopher Gunter, Katarina Matic, Mehrzad Sasanpour, Jennifer L. Ross, Parag Katira, Megan T. Valentine, Rae M. Robertson‐Anderson

**Affiliations:** ^1^ Department of Natural Sciences Scripps and Pitzer Colleges Claremont CA 92110 USA; ^2^ W. M. Keck Science Department Claremont McKenna College Claremont CA 91711 USA; ^3^ Department of Mechanical Engineering San Diego State University San Diego CA 92182 USA; ^4^ Department of Physics and Biophysics University of San Diego San Diego CA 92110 USA; ^5^ Department of Physics Syracuse University Syracuse NY 13244 USA; ^6^ Department of Mechanical Engineering University of California Santa Barbara CA 93106 USA

**Keywords:** active matter, composite, cytoskeleton, emergence, optical tweezers microrheology

## Abstract

The cytoskeleton is an active composite of filamentous proteins that dictates diverse mechanical properties and processes in eukaryotic cells by generating forces and autonomously restructuring itself. Enzymatic motors that act on the comprising filaments play key roles in this activity, driving spatiotemporally heterogeneous mechanical responses that are critical to cellular multifunctionality, but also render mechanical characterization challenging. Here, we couple optical tweezers microrheology and fluorescence microscopy with simulations and mathematical modeling to robustly characterize the mechanics of active composites of actin filaments and microtubules restructured by kinesin motors. It is discovered that composites exhibit a rich ensemble of force response behaviors–elastic, yielding, and stiffening–with their propensity and properties tuned by motor concentration and strain rate. Moreover, intermediate kinesin concentrations elicit emergent mechanical stiffness and resistance while higher and lower concentrations exhibit softer, more viscous dissipation. It is further shown that composites transition from well‐mixed interpenetrating double‐networks of actin and microtubules to de‐mixed states of microtubule‐rich aggregates surrounded by relatively undisturbed actin phases. It is this de‐mixing that leads to the emergent mechanical response, offering an alternate route that composites can leverage to achieve enhanced stiffness through coupling of structure and mechanics.

## Graphical abstract

Optical tweezers microrheology and fluorescence microscopy is coupled with simulations and mathematical modeling to discover that active cytoskeleton composites exhibit a rich ensemble of emergent mechanical response characteristics, from yielding to stiffening, governed by kinesin‐driven restructuring from well‐mixed interpenetrating double‐networks of actin and microtubules to de‐mixed states of microtubule‐rich aggregates surrounded by relatively undisturbed actin phases.

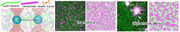

## 1 Introduction

The cytoskeleton is an active composite network of filamentous proteins and their associated binding proteins, including energy‐transducing molecular motors that pull and walk along filaments.^[^ ^1^, ^2^ ^]^ A primary role of the cytoskeleton is to provide mechanical integrity to cells while also allowing them to stiffen, soften, change shape, and generate forces, often in response to local stimuli.^[^ ^3^, ^4^ ^]^ These diverse mechanical responses are often spatially heterogeneous and can range from nanoscopic to cell‐spanning scales. Moreover, the nature of the response is intricately linked to the time‐evolving structures and interactions of the different networks of e.g., semiflexible actin filaments and microtubules.^[^ ^5^ ^]^

This complex active and composite nature of the cytoskeleton has rendered it a foundational model system for probing questions in active matter physics and addressing design challenges in living materials.^[^ ^3^, ^6^ ^]^ In vitro, cytoskeleton‐based active matter systems^[^ ^7^, ^8^, ^9^, ^10^, ^11^, ^12^, ^13^, ^14^, ^15^ ^]^ typically include myosin II minifilaments^[^ ^16^ ^]^ and/or crosslinked clusters of kinesin dimers,^[^ ^17^ ^]^ which are enzymatically‐active motor proteins that harness the energy of ATP hydrolysis to bind to and pull on actin filaments and/or microtubules, respectively. Actomyosin networks have been shown to undergo bulk contraction, local contraction into foci or asters, or disordered flow depending on the concentrations of the myosin, actin, and crosslinkers.^[^ ^12^, ^13^, ^18^ ^]^ Kinesin clusters acting on bundles of microtubules have also shown varied behaviors, ranging from the formation of locally condensed asters^[^ ^19^, ^20^, ^21^, ^22^, ^23^ ^]^ to space‐spanning networks capable of extensile restructuring that results in nematic flow and organization reminiscent of liquid crystals.^[^ ^8^, ^24^, ^25^ ^]^

More recently, in vitro active cytoskeletal composites that include both actin and microtubules have been engineered and examined,^[^ ^26^, ^27^, ^28^, ^29^, ^30^, ^31^, ^32^, ^33^ ^]^ often revealing emergent behavior and improved material properties, such as organized dynamics,^[^ ^26^ ^]^ tunable miscibility,^[^ ^30^, ^33^ ^]^ structural memory,^[^ ^31^ ^]^ and enhanced elasticity.^[^ ^28^ ^]^ Early work explored passive composite networks lacking molecular motors. In this simplified condition, biotin‐streptavidin crosslinkers induce passive and effectively permanent crosslinking of either the actin or microtubule components.^[^ ^34^, ^35^ ^]^ Within such composites, microtubule crosslinking is essential to eliciting elastic responses to localized strains, whereas actin‐crosslinked composites exhibit yielding behavior similar to that of purely entangled composites.^[^ ^34^ ^]^

When the concentration of actin crosslinkers is varied, an emergent elasticity is revealed at intermediate crosslinker:actin ratios R≃0.2, which decreases to values comparable to those of entangled composites as this ratio is increased to R≃0.8.^[^ ^35^ ^]^ This counter‐intuitive behavior is observed only in composites, and is driven by crosslinker‐mediated network coarsening and bundling that simultaneously increases the thickness (and thus stiffness) of network fibers and as well as network mesh size. The delicate interplay of actin network microstructure and fiber rigidity leads to the development of an optimal crosslinker ratio in which the network has developed sufficient rigidity to maximize elastic response, but the mesh size remains small enough to suppress the diffusive mobility of microtubules entrapped within the actin network.^[^ ^35^ ^]^ Notably, for actin‐only networks, increasing crosslinker ratios monotonically increases the network stiffness.^[^ ^36^, ^37^, ^38^ ^]^ However, within the composites, the increased microtubule mobility enables new pathways of stress relaxation, which dominate the mechanical response and soften and fluidize the composite.

When enzymatically‐active motors are included, even more dramatic structural changes are observed. Embedded motors act both as transient crosslinkers and force‐generating elements, leading to dynamic responses that span spatial and temporal scales. Co‐entanglement of microtubules with myosin‐driven actin filaments produces active composite networks in which both actin and microtubules ballistically contract at speeds that can be tuned by the concentrations of actin and myosin.^[^ ^26^, ^27^ ^]^ Such networks display controlled motion, enhanced elasticity, and sustained structural integrity as compared to single‐filament networks.^[^ ^28^ ^]^ Replacing myosin with kinesin as the active agent in similar co‐entangled composites results in a much higher degree of variability in dynamics and structure, with speeds that vary by 2 orders of magnitude, phases of acceleration and deceleration, and enhanced restructuring and de‐mixing of actin and microtubules, depending on the composite formulation and time after motor activation.^[^ ^33^ ^]^ Typically, network restructuring occurs over a finite timespan that is determined by the motor‐driven compaction of filaments into poorly‐connected, kinetically trapped network structures reminiscent of asters or disordered aggregates, with kinesin‐driven composites displaying shorter active lifetimes as compared to those driven by myosin or both myosin and kinesin.

Similar phenomena have been observed in composite networks comprising kinesin clusters and microtubules that are bundled by osmotic crowders acting as depletants and by microtubule‐binding proteins that promote antiparallel bundling.^[^ ^30^ ^]^ Adding low concentrations of actin to such networks produces fluid‐like extensile dynamics, similar to those of kinesin‐driven microtubule networks lacking actin. However, when the actin concentration is increased, rich dynamic structural transitions are observed, leading to the formation of onion‐like asters of layered actin and microtubules or bulk contractility. Further increases in actin concentration promote de‐mixing of actin and microtubules with asters and contractile regions becoming increasingly microtubule‐rich.^[^ ^30^ ^]^

Motor‐driven structural transitions that form disconnected, filament‐dense structures interspersed within a dilute fluid phase can undermine network percolation. From a material design perspective, this phase segregation risks fluidizing the network on mesoscopic scales, thereby compromising the ability of active composites to transmit forces over large distances or to sustain significant external stresses. Despite the importance of the mechanical response of these systems to their role in cellular processes and to materials‐based applications, the majority of active composite studies have focused on the evolving structure and dynamics of the materials without regard to mechanical properties.

While there are a number of approaches to analyzing network structural rearrangements via fluorescence microscopy, the measurement of the time‐evolving local mechanical properties within the dynamically‐restructuring network remains technically challenging. The emergent heterogeneity arising due to motor‐driven aggregation or segregation of filaments demands the precise application of forces to determine the local mechanical responses,^[^ ^28^ ^]^ as well as relatively large numbers of measurements in order to develop an understanding of average responses and the range of variations at each condition. Thus, although a key feature of motor‐driven active cytoskeletal composites is their ability to flow, coarsen, and reconfigure due to internal motor‐generated forces, little is known about the interplay between material mechanics and filament motion during the emergence of these new structural phases.

To begin to establish the foundational role of motor‐driven restructuring in network mechanics, we designed co‐entangled composites of actin and microtubules formulated to support robust connectivity, and subjected them to active stresses and restructuring by adding varying concentrations of kinesin motors. Using a comprehensive platform comprising an optical tweezers microrheometer (OTM) capable of applying large‐scale strains at specified locations within the heterogeneous sample, fluorescence microscopy to assess structural rearrangements, simulations based on lattice‐based advection‐diffusion models, and mathematical modeling of mechanical responses, our results reveal how kinesin motors act on composites of actin and microtubules to sculpt the mechanical and structural properties across spatiotemporal scales. We identify the presence of kinesin‐driven demixing via clustering, which in turn leads to emergent complexity in mechanical response and formulation‐dependent heterogeneity that can be captured both in vitro and in silico. These results demonstrate the importance of hierarchical structural heterogeneity to provide new avenues for enhanced stiffness and relaxation only possible in composite designs.

## 2 Results and Discussion

### 2.1 Kinesin Motors Drive De‐Mixing via Clustering of Microtubules

**Figure 1:**
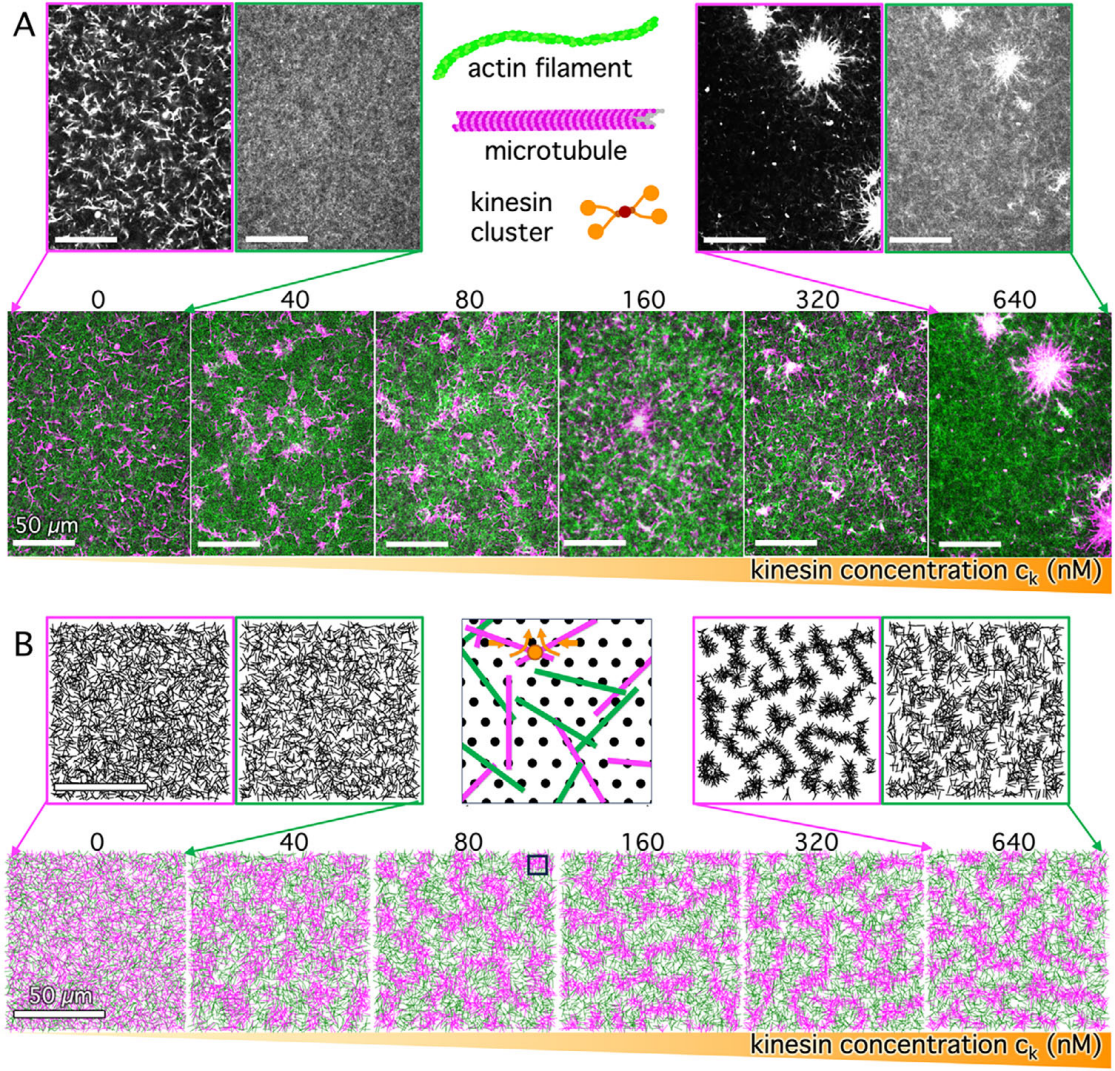
Kinesin motors drive de‐mixing of co‐entangled actin and microtubules. A) Two‐color fluorescence confocal microscopy images of composites of microtubules (magenta) and actin filaments (green) in the presence of varying concentrations of kinesin motors, listed in nm above each composite image. Greyscale images show separate channels for microtubules (magenta borders) and actin (green borders) for $c_{k}=$ 0 nm (left) and $c_{k}=$ 640 nm (right). All scale bars denote 50 µm. Schematics show composite components (not to scale). B) Snapshots from simulations of 2D kinesin‐driven composites of actin and microtubules with the same effective kinesin concentrations as in experiments. Colors and labels are the same as in (A). Center schematic is a zoom‐in of the simulated composite with black circles denoting lattice points that can be occupied (or not) by either a single microtubule (magenta) or single actin filament (green). The orange circle and arrows denote, respectively, a kinesin motor and filament rotation (curved arrows) and contraction (straight arrows) that it can impart on microtubules. The scale of the schematic is indicated by the black box in the upper right corner of the $c_{k}=$ 80 nm snapshot.

As a first step, we assess the complex structural properties of active composites composed of actin, microtubules, and kinesin. We judiciously chose a ratio of actin to microtubules (45:55 molar ratio of actin to tubulin dimers) that allows for active restructuring and force‐generation without the large‐scale flow or network rupturing that has been previously reported,^[^ ^30^, ^33^ ^]^ and examined the effect of varying concentrations of kinesin, $c_{k}$, on the network restructuring (**Figure** **1**A). In control networks lacking kinesin, we observed, using high‐resolution two‐color confocal microscopy, uniform mixing of actin and microtubules, to form a homogeneous, space‐spanning composite of interpenetrating networks of actin and microtubules (Figure 1A, left). Upon addition of kinesin, we observed the formation of microtubule‐rich phases that appeared to generally increase in size, density, and number with increasing kinesin concentration. This kinesin‐driven de‐mixing of microtubules from actin was robustly observed across all samples; and, upon addition of 640 nm kinesin, the highest concentration investigated here, nearly all of the microtubules condense into aster‐like aggregates surrounded by actin‐rich zones (Figure 1A, right).

To better understand the molecular drivers of this behavior, we build on our previously developed 2D lattice‐based advection‐diffusion model of filament dynamics.^[^ ^33^ ^]^ Within the model, filament motion arises from kinesin‐generated active forces that can either pull or push microtubules, as well as frictional forces that occur when passive motors act as crosslinkers between microtubules.^[^ ^33^ ^]^ In the control case without kinesin, the simulations rendered a uniform, well‐mixed composite of interpenetrating networks of microtubules and actin, consistent with our experimental observations (Figure 1B, left). Similarly, upon addition of kinesin motors, the composites restructure and de‐mix, with increasing segregation observed for higher kinesin concentrations. Moreover, it is clear from both experiments and simulations that the kinesin‐driven motions that cluster the microtubules do not significantly restructure the actin. Rather, a two‐phase material is formed, with microtubule‐dense regions forming distinct, well‐separated aggregates within a more uniform actin‐rich background (Figure 1B, right).

**Figure 2:**
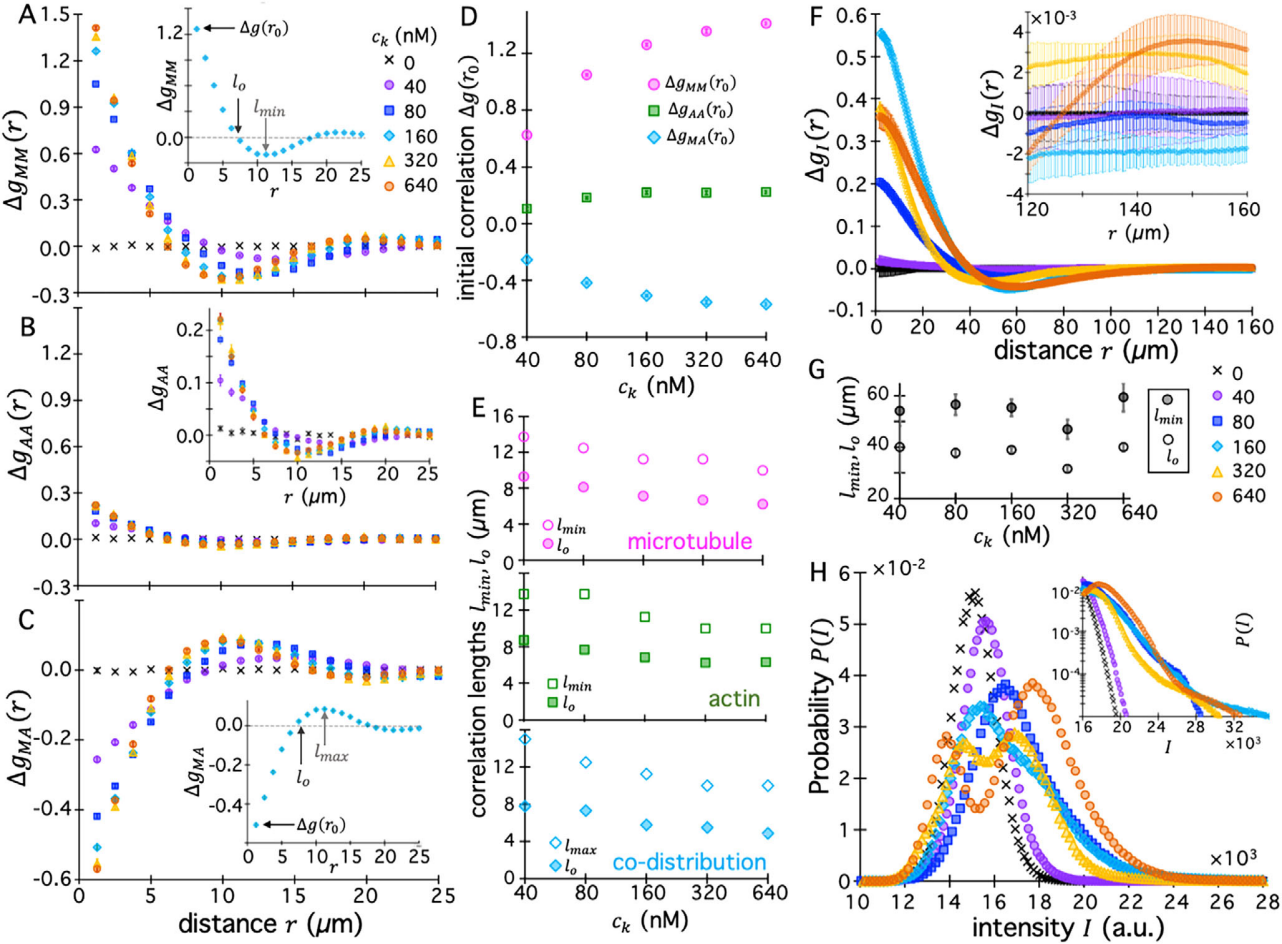
Filament pair correlations and clustering of microtubules increase with increasing motor concentration over varying lengthscales. A–C) Correlation analysis of simulated composites at varying kinesin concentrations (listed in legend in A) shows increased correlation between (A) microtubule pairs $\Delta g_{MM}\left(r\right)$ and (B) actin pairs $\Delta g_{AA}\left(r\right)$ as well as (C) decreased co‐distribution of actin and microtubules at short distances r compared to the no kinesin case ($c_{k}=$ 0, black × markers). Insets in (A,C) depict metrics plotted in (D,E). Inset in B is zoom‐in of $\Delta g_{AA}\left(r\right)$. D) Initial values of distributions plotted in (A–C), $\Delta g_{MM}\left(r_{0}\right)$ (magenta circles), $\Delta g_{AA}\left(r_{0}\right)$ (green squares), and $\Delta g_{MA}\left(r_{0}\right)$ (blue diamonds), show increasing like‐filament correlation and decreasing co‐distribution as $c_{k}$ increases. (E) Correlation lengths determined as the radial distances r at which $\Delta g_{MM}\left(r\right)$ (top, magenta), $\Delta g_{AA}\left(r\right)$ (middle, green) and $\Delta g_{MA}\left(r\right)$ (bottom, blue) reach zero ($l_{0}$, filled symbols) and local extrema ($l_{min}$, $l_{max}$, open symbols). F,G) Spatial image autocorrelation analysis of experiment videos of labeled filaments, showing the (F) average autocorrelation difference of pixel intensities $\Delta g_{I}\left(r\right)$ for varying kinesin concentrations (see legend below), and (G) the corresponding correlation lengths $l_{0}$ (open symbols) and $l_{max}$ (filled grey symbols) versus $c_{k}$. Inset in (F) shows zoom‐in of $\Delta g_{I}\left(r\right)$ curves at large distances with error bars (which are too small to see in the main plot) denoting standard error. H) Probability distributions of pixel intensities P(I) for varying kinesin concentrations. Inset shows distributions on a semi‐log scale to better visualize the high‐intensity tails at high kinesin concentrations ($c_{k}>$ 80 nm).

The simulations also provide the opportunity to quantitatively compare the simulated network structures before and after restructuring through calculation of the filament pair distribution function $g_{ij}\left(r\;,T\right)$ where the subscripts i and j represent the filament type, either actin (A) or microtubules (M), and which gives the probability of finding a filament (actin or microtubule) a radial distance r from any other filament. To assess the dynamic structural changes that occur within a single filament network during the simulation, we report the difference between these quantities for the initial (T= 0) state and final $\left(T=\;T_{F}\right)$ states: $\Delta g_{ii}\;\left(r\;\right)=g_{ii}\left(r\;,T_{F}\right)-\;g_{ii}\left(r\;,0\right)$. For static, steady‐state networks, we expect $\Delta g_{ii}\;\left(r\right)=$ 0 for all r, as we see in **Figure** **2**A,B for both the actin and microtubule networks within the composites lacking kinesin ($c_{k}=$ 0). This result also validates that our initial simulation conditions represent homogeneous, well‐mixed networks that remain well‐mixed in the absence of motor activity. When comparing the distribution of microtubules to other microtubules, or actin filaments to other actin filaments, we found positive values of $\Delta g_{MM}\left(r\right)$ and $\Delta g_{AA}\left(r\right)$ for small filament separation distances r, indicating attractive interactions that drive clustering on those length scales; and we observed an increased clustering propensity with increased kinesin concentration, estimated by the value of $\Delta g_{ii}\left(r_{0}\right)$, where $r_{0}=$1.25 µm is the smallest radial distance between lattice points in the simulation (Figure 2D). As the separation distance increases, $\Delta g_{ii}\left(r\right)$ curves for all $c_{k}>$ 0 composites decay to zero then continue to decrease, reaching local negative‐valued minima before asymptoting back to zero. This behavior indicates a depletion of filaments on intermediate‐length scales, which we interpret as an indication of clustering. While both filament types display these general features, the microtubule network, upon which the kinesin motors directly act, exhibit much stronger clustering effects compared to actin (Figure 2A,B,D).

By contrast, when evaluating the co‐distribution of actin filaments with respect to the microtubule network, we find negative values of $\Delta g_{MA}\left(r\right)$ at even the smallest filament separation distances (Figure 2C), indicating an exclusion of the unlike filament type. This anti‐correlation demonstrates that actin is displaced from the microtubule‐rich domains that form from the kinesin‐driven contraction of microtubules, and is consistent with de‐mixing. The strength of this effect can be approximated by $\Delta g_{MA}\left(r_{0}\right)$, which is found to monotonically decrease with increasing kinesin concentration (Figure 2D). The magnitude of this exclusion effect $|\Delta g_{MA}\left(r_{0}\right)|$ is intermediate between the values observed for clustering of the microtubule $\Delta g_{MM}\left(r_{0}\right)$ and actin $\Delta g_{AA}\left(r_{0}\right)$ structures. The length scales over which this phase separation was observed can be approximated by the radial distance at which $\Delta g_{ij}\;\left(r\right)=$ 0, which we denote as $l_{0}$, as well as the distance at which $\Delta g_{ij}\left(r\right)$ is minimal for like‐filament distributions or maximal for microtubule‐actin co‐distributions, which we denote by $l_{min}$ or $l_{max}$. Specifically, $l_{0}$ can be considered a measure of cluster size while $l_{min}$ and $l_{max}$ are measures of spacing between clusters. In a system with mass conservation, we expect both quantities to generally track with one another, as we observe in Figure 2E.

As shown in Figure 2E, we found similar length scales of de‐mixing when comparing all filament types, and, in each case, we observed a monotonic decrease in the observed length scales with increasing kinesin concentrations. Moreover, the range of values (≈5–15 µm) were generally consistent with the observed sizes and spacing between clusters in both experiment and simulation (Figure 1), and reflect the increased clustering with increasing kinesin concentration.

To more quantitatively compare these structural analysis results from simulations to experimentally observed restructuring, we performed spatial image autocorrelation (SIA) analysis^[^ ^28^, ^39^ ^]^ on epifluorescence movies of the composites captured in identically prepared samples as the force measurements that we describe below. In SIA, the correlation in intensities between two pixels separated by a distance r is examined. In our experiment, both actin and microtubules were labeled with spectrally‐indistinguishable fluorescent dyes and were simultaneously imaged such that at each condition, the composite network behavior is observed (see Methods). Similar to pair distribution functions obtained from simulations, the resulting autocorrelation function $g_{I}\left(r\right)$, decays from a maximum value at $r_{0,I}=$ 0.41 µm (set by the pixel size) to $g_{I}=$ 0 as r→∞, passing through a local $g_{I}<$ 0 minimum; and at a given distance larger $g_{I}\left(r\right)$ values are suggestive of increased filament clustering. To better compare the simulated distributions shown in Figure 2A–C to experimentally determined data, we subtracted the value of $g_{I}\left(r\right)$ obtained for the composite without kinesin ($c_{k}=$ 0) from $g_{I}\left(r\right)$ for each $c_{k}>$ 0 composite, similar to subtracting the initial time distribution from the final in simulations. As shown in Figure 2F, the resulting $\Delta g_{I}\left(r\right)$ curves display similar functional features as the simulated data, with higher kinesin concentrations generally resulting in larger $g_{I}\left(r_{0}\right)$ values and more pronounced minima. However, a striking distinction is that for experiments, $g_{I}\left(r_{0}\right)$ displays a non‐monotonic dependence on $c_{k}$, reaching a maximum for $c_{k}=$ 160 nM. This non‐monotonicity is reminiscent of similar emergent phenomenon reported for cytoskeleton composites with increasing concentrations of actin crosslinkers.^[^ ^35^ ^]^

Evaluating the same characteristic distances as in simulations for cluster size and spacing, $l_{0}$ and $l_{min}$, namely where $\Delta g_{I}\left(r\right)$ reaches zero and a local miminum, we find that both lengthscales show a modest decrease between $c_{k}=$ 40 nm and $c_{k}=$ 320 nm, similar to simulations, but subsequently increases at $c_{k}=$ 640 nm (Figure 2G). This increase is indicative of the large aggregates we observe in microscopy images (Figure 1A). We note that the correlation lengthscales observed in experimental data (≈30–60 µm) are generally larger than for simulations, likely due to the larger field‐of‐view and system size, as well as the added dimension in 3D experiments. Specifically, the 2D experimental plane is ≈10^3^‐fold larger than our simulation box size, so we have many more filaments able to move into the imaging field‐of‐view to join clusters. Conversely, once most simulated filaments end up in clusters, and there are few freely diffusing filaments left in the box, the clusters cannot grow further. The third dimension in experiments provides another route for filaments to move and reorganize to facilitate cluster growth.

Further examining the large lengthscales accessible to experiments, we observed positive $g_{I}\left(r\right)$ values out to the largest analyzed distance ($r_{\infty }=$ 160 µm) for the highest kinesin concentrations ($c_{k}=$ 320, 640 nm), indicative of the presence of large‐scale clustering in these conditions (Figure 2F, inset). By contrast, the $c_{k}=$ 160 nm composite, which displayed the highest short‐range correlation, exhibited negative long‐range correlation values as $r\to r_{\infty }$. Together, these data suggest that as the kinesin concentration increases, de‐mixing initially causes dense small‐scale clustering, as indicated by the peak in $g_{I}\left(r_{0}\right)$ at intermediate kinesin concentration, followed by large‐scale phase separation that maximizes $g_{I}\left(r_{\infty }\right)$ for higher kinesin concentrations.

To further corroborate the physical picture of de‐mixing, we also examined the distribution of pixel intensities of the same videos. Using intensity as a proxy for mass, we evaluated the distribution of pixel intensities to identify increases (higher pixel values) and decreases (lower pixel values) in filament density due to bundling and clustering (Figure 2H). We found that as the kinesin concentration was increased from $c_{k}=$ 0 nm to 80 nm, the peaks of the distribution shifted to higher intensity values, indicating bundling; and the distributions became broader, indicating the increasingly heterogeneous distribution of densities. For $c_{k}\geq$ 160 nm, two peaks emerged. The higher intensity peak occurred at an intensity that was slightly larger than that of the single peak for the $c_{k}<$ 160 nm conditions, and this peak shifted to higher intensity values (shifting further right) and larger probabilities (increasing height) as $c_{k}$ increased. This trend is indicative of an increasing number of bundles that also become denser due to the presence of additional kinesin motors. The second peak, which occurred at lower intensity values than the single peaks observed for $c_{k}<$ 160 nm, likewise shifted to lower intensity values as $c_{k}$ increased, indicating the emergence of more microtubule‐poor zones. Zooming‐in to examine the high‐intensity tails of the distributions, we found that composites with $c_{k}\geq$ 160 nm exhibited pronounced extended tails that were not observed for the lower kinesin concentrations, again indicating the formation of large and dense clusters at the higher concentrations of kinesin (Figure 2H, inset).

Together, these results indicate that kinesin clusters drive contraction and compaction of disordered microtubules into dense, well‐separated aggregates. This contraction occurs in the absence of osmotic crowding agents and does not require the presence of non‐motor microtubule‐associated binding proteins to promote bundling. This restructuring causes modest reorganization of the actin network, as the actin filaments are squeezed out by contracting microtubules. However, the actin network remains reasonably well dispersed even at the highest kinesin concentrations.

### 2.2 De‐Mixing Drives Emergent Complexity in Mechanical Response

**Figure 3:**
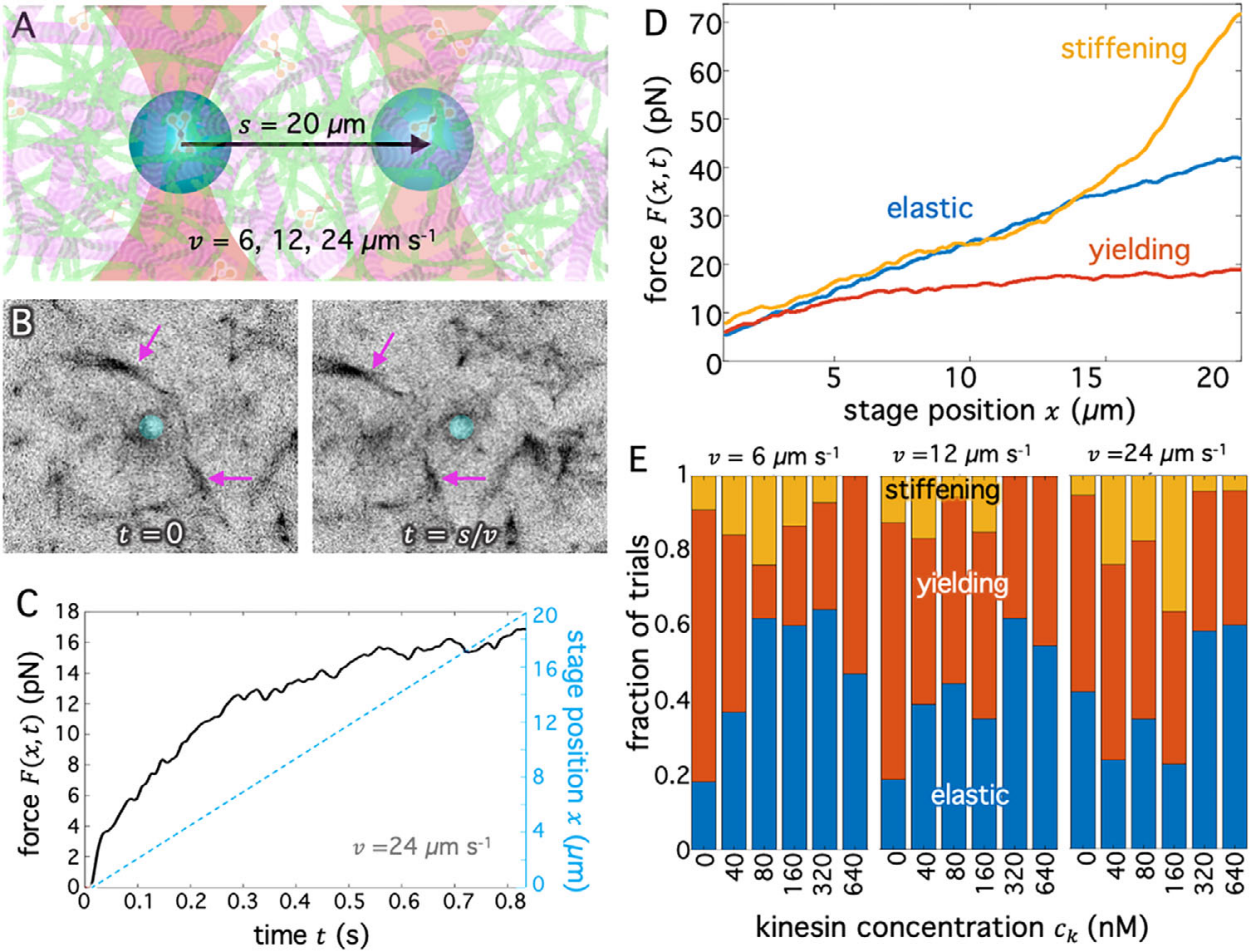
Optical tweezers microrheology reveals heterogeneous distribution of force responses of kinesin‐driven composites to local mesoscopic strains. A) Schematic showing a focused infrared laser (red) trapping a probe particle of diameter 4.5 µm (blue) embedded in a kinesin‐driven actin‐microtubule composite. The sample is deformed locally by the application of a constant‐speed strain operating over a distance s= 20 µm at speeds v= 6, 12, 24 µm s^−1^, by the action of a piezoelectric stage moving the sample relative to the trap. B) Inverted greyscale images of labeled fibers in the composite being displaced (magenta arrows) as the stage moves relative to the fixed trap that holds the probe (highlighted in cyan). The images show the time immediately before (t= 0, left) and after ($t=\frac{s}{v}\;$, right) the initial stage sweep; magenta arrows highlight structures visible in both images to demonstrate the relative motion of the trapped particle with respect to the surrounding composite. C) Example of the force (black) exerted on the probe versus time t in response to the stage (sample) moving through a displacement (cyan, dotted line) of s= 20 µm relative to the trap at a speed of v= 24 µm s^−1^. D) Representative examples of force‐displacement curves demonstrating the three classes of responses that composites exhibit: elastic (blue), yielding (orange), and stiffening (gold). E) Fraction of trials that exhibited each response class, color‐coded as in panel D, for all six kinesin concentrations (x axis) and all three speeds: 6 µm s^−1^ (left), 12 µm s^−1^ (middle), and 24 µm s^−1^ (right). The total number of measurements per condition varied from 11 to 25.

To probe how the kinesin‐driven restructuring influences the microscale mechanical properties of the composite, we applied localized but large‐scale deformations within the heterogeneous material using an optical trapping‐based manipulation platform (**Figure** **3**A,B).^[^ ^40^, ^41^, ^42^ ^]^ A single‐beam gradient optical trap was formed by tightly focusing a high‐powered IR laser to a diffraction‐limited volume within the sample chamber,^[^ ^43^ ^]^ allowing the capture and manipulation of embedded colloidal probes (radius $r_{p}=$ 2.25 µm, full details provided in Experimental Section). The trap stiffness, which was calibrated through independent measurements, was sufficient to stably trap and hold the particle, even as the stage moved at fixed velocity, thereby dragging the particle through the sample. The displacement of the trapped particle from the trap center was simultaneously monitored in real time, and when multiplied by the known trap stiffness, provided an instantaneous readout of the force. From the known values of force and stage position, which are collected as a function of time (Figure 3C), it is possible to construct a relationship between force and stage position. Thus, this instrument acts as a microscale mechanical testing system or microrheometer, which we use to interrogate the response to a strain (i.e., stage displacement) of s= 20 µm, which we chose to be significantly larger than the probe size $r_{p}$ and composite mesh size ξ≃ 1.2 µm (see Experimental Section). We performed measurements on composites with the six kinesin concentrations presented above (Figures 1 and 2), using three stage speeds (v= 6, 12, 24 µm s^−1^) for each concentration.

Examining the individual force‐displacement traces (Figure 3C,D), we find that all traces exhibited a sharp initial increase in force at the smallest stage displacements as the particle position rapidly shifted within the trap as the stage began to move. The time constant associated with this re‐equilibration is given by the ratio of local drag coefficient to the trap stiffness $k_{OT}$ and was typically <50 ms. Beyond this very initial behavior, we find heterogenous responses, which can be categorized into three broad classes of responses (shown in representative traces in Figure 3D): traces that are fully linear, suggesting elastic behavior (‘elastic’), those that show an initial elastic response that softens or yields over time (‘yielding’), and those that show an initially soft elastic response that significantly stiffens at large displacements (‘stiffening’). The proportion of traces falling into each category varies as a function of kinesin concentration and stage speed (Figure 3E). In general, the responses are diverse and heterogeneous, likely reflecting the structural heterogeneity of the material observed in Figures 1 and 2. The observance of a large fraction of fully linear elastic traces is notable, given the stage stroke of 20 µm, an order of magnitude larger than the size of both the probe and mesh.

**Figure 4:**
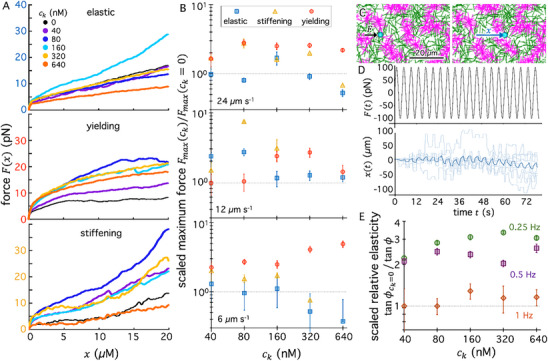
Force response of kinesin‐driven composites displays non‐monotonic dependence on kinesin concentration. A) Average force F(x) versus stage position x measured in response to v= 24 µm s^−1^ straining, for each kinesin concentration $c_{k}$, listed and color‐coded according to the legend in the top panel. Force traces classified as elastic (top, blue y‐axis), yielding (middle, red y‐axis), and stiffening (bottom, gold y‐axis) are averaged separately. Responses at v= 6 µm s^−1^ and v= 12 µm s^−1^ are shown in Figure S2 (Supporting Information). B) Maximum force reached during the strain $F_{max}$, determined from average force traces, and normalized by the corresponding value at $c_{k}=$ 0 (denoted by the dashed horizontal line), for each class of response: elastic (blue squares), yielding (red circles), stiffening (gold triangles). Data plotted in the top panel correspond to the curves shown in A. Middle and bottom panels are for v= 12 µm s^−1^ and v= 6 µm s^−1^. Error bars correspond to standard error. C) Sample simulated composite with embedded 1 µm particle (cyan) subject to force F (black arrow, left) that displaces the particle a distance x (blue arrow, right). D) Simulated strains are sinusoidal with amplitude of $F_{0}=$ 100 pN, and result in oscillatory particle displacements (light blue) which are averaged together (blue) to determine viscoelastic moduli $G^{\prime }$ and $G^{\prime \prime }$ by evaluating the phase shift ϕ between F and x. Sample data shown is for $c_{k}=$ 160 nm and oscillation frequency of 0.25 Hz. E) Scaled relative elasticity, computed as the inverse loss tangent ${\left[\tan\phi \right]}^{-1}$ normalized by the corresponding $c_{k}=$ 0 value, indicated by the dashed horizontal line, versus kinesin concentration for strain frequencies of 0.25 Hz (green circles), 0.5 Hz (purple squares) and 1 Hz (red diamonds). Error bars correspond to standard deviation of bootstrapped ensembles.

To better assess the dependence of mechanical properties on composite formulation, we analyzed the force‐displacement traces measured for >20 different particles in different locations and samples for each experimental condition (see Methods). For each combination of kinesin concentration $c_{k}$ and speed v, we averaged together all traces that displayed elastic, yielding, or stiffening characteristics (**Figure** **4**A; Figure S2, Supporting Information). For each of these response types, we observed striking nonmonotonic behavior as the kinesin concentration was increased.

When we examined the subset of elastic traces obtained at v= 24 µm s^−1^, we found that the largest value of maximum force and maximum effective stiffness, as qualitatively assessed from the terminal force value $F_{max}\;$ reached at the end of the strain, occurred at the intermediate value of $c_{k}=$ 160 nm (Figure 4A,B). The lowest values of $F_{max}$ were surprisingly observed at the highest concentration of $c_{k}=$ 640 nm. Similar general trends were observed for the elastic traces obtained at the other speeds (Figure 4B; Figure S2, Supporting Information).

For yielding traces observed at v= 24 µm s^−1^, the measured force typically settled to a plateau value for stage displacement values above ≈5 µm (Figure 4A), which corresponds to a local strain of ≈1, if we estimate the local strain by normalizing the stage displacement by the trapped particle diameter.^[^ ^40^, ^45^ ^]^ The maximum force value initially increased with increasing concentrations of kinesin until the intermediate value of $c_{k}=$ 80 nm was reached, after which the $F_{max}$ showed a slight decline, as shown in Figure 4B where we display $F_{max}$ normalized by the $c_{k}=$ 0 nm value. Similar trends were observed for the yielding traces obtained at v= 12 µm s^−1^, whereas at v= 6 µm s^−1^, $F_{max}$ increased monotonically with increasing concentrations of kinesin (Figure 4B). Among the particles exhibiting stiffening at v= 24 µm s^−1^, we observed a peak in maximum force at $c_{k}=$ 80 nm, which then decreased to a value lower than that of the initial $c_{k}=$ 0 nm condition at the largest concentration of $c_{k}=$ 640 nm (Figure 4A,B). Similar trends were observed for the stiffening traces obtained at the other speeds (Figure 4B; Figure S2, Supporting Information).

Two notable takeaways from these results are that 1) active composites exhibit emergent mechanical resistance at intermediate kinesin concentrations, and 2) the viscoelastic response of composites to the application of local strains is heterogeneous, varying from stiffening to elastic to more viscous‐dominated (i.e., yielding).

To shed further light on these features, and assess the ability of our simulations to capture them, we introduced spherical probes into our simulated composites and imparted oscillatory forcing on them through the composite (Figure 4C). As described in Methods and SI, we measured the probe displacement resulting from oscillatory forcing with amplitude $F_{0}=$ 100 pN and frequencies ω= 0.25, 0.5, and 1 Hz, to determine the viscoelastic stress response (Figure 4D). We chose the force amplitude to achieve particle displacements comparable to our 20 µm experimental strain (Figure 4D), and frequencies to approximately match those in our experiments, considering ω=v/s. Specifically, we measured the bead displacement amplitude and phase difference ϕ between the oscillation in applied force and resulting bead displacement to determine the elastic modulus $G^{\prime }$ and viscous modulus $G^{\prime \prime }$ as a function of kinesin concentration (Figure S3, Supporting Information). To quantify the relative elasticity of the composite, we evaluated the inverse loss tangent ${\left[\tan\phi \right]}^{-1}=G^{\prime }/G^{\prime \prime }$ which is increasingly >1 or <1 for more elastic‐dominated or more viscous‐dominated responses, respectively. We found that introducing kinesin into composites increased the relative elasticity for all frequencies (Figure 4E), and that peak elasticity was observed at intermediate kinesin concentrations for 0.25 and 1 Hz. The 0.5 Hz data also showed a local maximum at intermediate kinesin concentrations but then increased again at $c_{k}=$ 640 nm. These features align with our experimental results that display non‐monotonic dependence of the force response on $c_{k}$ for most formulations and speeds; and highlight the importance of both elastic and viscous contributions to the force response. Collectively, these results demonstrate the emergent elasticity and force resistance that kinesin‐driven de‐mixing affords, which is optimized at intermediate kinesin concentrations.

**Figure 5:**
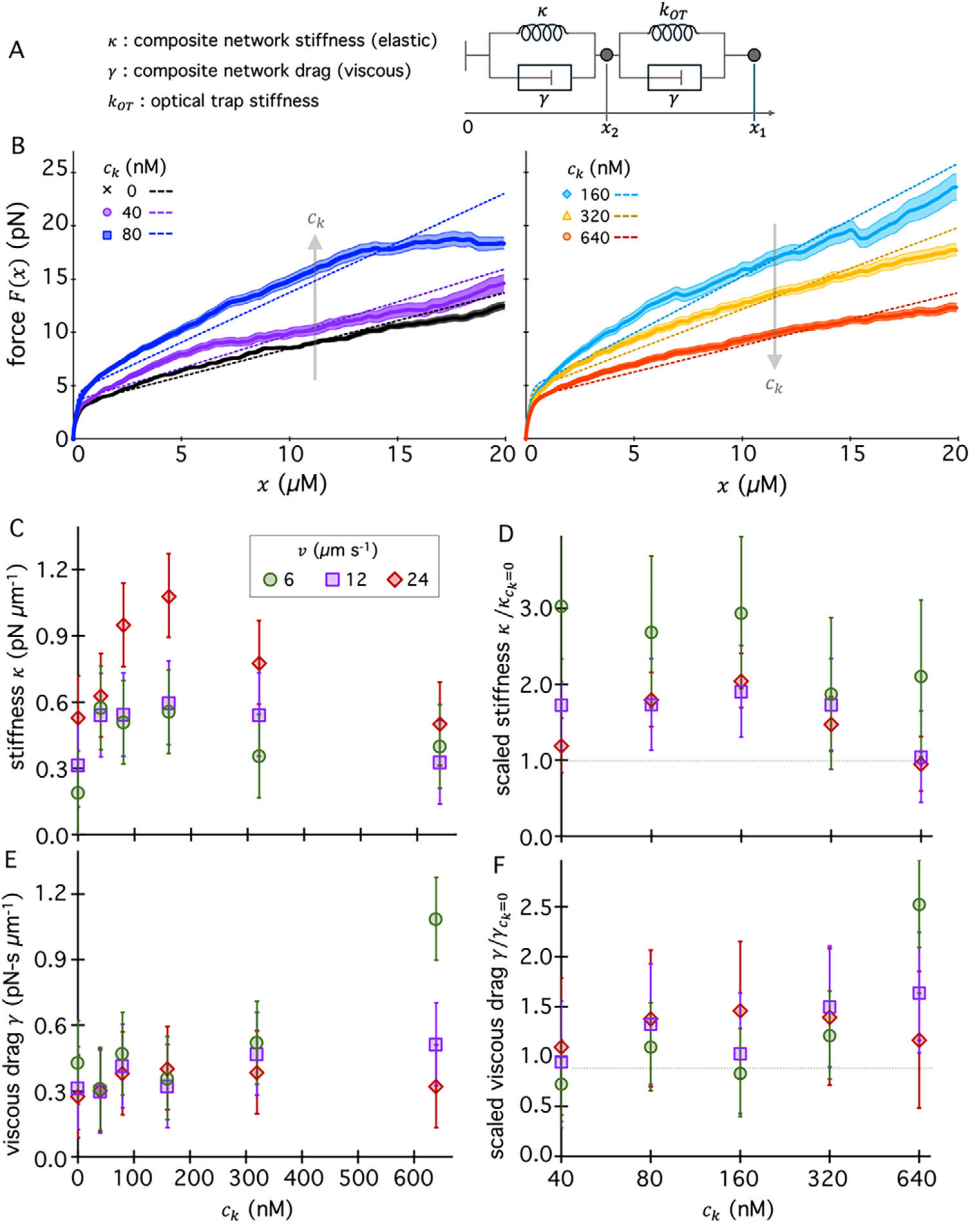
Mechanical circuit model captures the viscoelastic behavior of elastic and yielding response classes and emergent stiffness at intermediate kinesin concentrations. A) Cartoon of mechanical circuit that models the force‐displacement relationship for a bead pulled through a network by an optical trap with known trap stiffness $k_{OT}$. The composite network stiffness κ and drag γ are fit parameters in the model. B) Force F(x) versus stage position x, averaged across all elastic and yielding traces for each kinesin concentration $c_{k}$, listed and color‐coded according to the legend, for v= 24 µm s^−1^. Error bars denote standard error of the mean. Dashed lines are fits to the equation of motion for the mechanical circuit depicted in (A). (C‐F) Fit parameters κ (C,D) and γ (E,F) as a function of kinesin concentration $c_{k}$ for speeds v= 6 (green circles), 12 (purple squares), and 24 (red diamonds) µm s^−1^, with error bars denoting 95% confidence intervals. Panels display (C,E) magnitudes for all kinesin concentrations on a linear scale and (D,F) values for $c_{k}>$ 0 normalized by their corresponding $c_{k}=$ 0 value and shown on a log scale.

To more quantitatively understand the tunable viscoelastic nature of the experimental force responses, and their underlying drivers, we use a mechano‐equivalent circuit approach to model the ensembled‐averaged responses.^[^ ^46^, ^47^ ^]^ Due to the relative infrequency of the stiffening responses, and the likelihood that this subset of traces is dominated by rare interactions of the particles with heterogeneous microstructures, we focused our analysis on the elastic and yielding responses only. We designed an equivalent circuit that consists of two Kelvin–Voigt elements in series (**Figure** **5**A, details in Supporting Information). The first element accounts for the composite network viscoelasticity, which is represented by a spring element with spring constant κ to represent the network stiffness, and a dashpot element with drag coefficient γ to represent viscous dissipation. A second Kelvin–Voigt element represents the effect of the optical trap stiffness $k_{OT}$ (which is known). We allowed the two elements to undergo relative deformation, and tracked the position of the center of the optical trap and the particle as $x_{1}$ and $x_{2}$, respectively. Here, $x_{1}=vt,$where v is the stage speed and t is the elapsed time. To estimate the force response as a function of stage motion, we calculate $F=k_{\mathrm{OT}}\;\left(x_{1}-x_{2}\right).$ Assuming that $x_{1}=x_{2}\;\approx 0$ at t=0, we established:

(1) $$F\;\left(x_{1}\right)=\frac{k_{\mathrm{OT}}\kappa }{k_{\mathrm{OT}}+\kappa }\cdot x_{1}-\gamma vk_{\mathrm{OT}}\left(\frac{\kappa -k_{\mathrm{OT}}}{{\left(k_{\mathrm{OT}}+\kappa \right)}^{2}}\right)\left(1-e^{-\left(\frac{k_{\mathrm{OT}}+\kappa }{2\gamma }\right)x_{1}}\right)$$

We selected this model to capture the following phenomena: an initial elastic jump due to the re‐equilibration of the particle within the optical trap as the stage begins to move, the transition to a second elastic regime as the particle engages with the composite network, and the presence of transient bonds that can dissipate stress and can be modeled via an effective viscosity (Figure 5B). We use this approach to analyze data for each of the six kinesin concentrations at each of the three tested stage speeds.

For each speed, we found a non‐monotonic dependence of κ on $c_{k}$, with the highest stiffness observed at $c_{k}=$ 160 nm (Figure 5C,D), consistent with both our force analysis (Figure 4), as well as our structural assessments that showed a higher propensity for microtubule crosslinking and small‐scale bundling at intermediate kinesin concentrations (Figure 2). Additionally, we found that the highest stiffnesses occurred at the fastest stage speeds, which may reflect the reduced ability for the network to relax on the timescale over which the strain is applied. Specifically, the presence of semiflexible actin filaments in the composites allows for actin bending modes to dissipate stress.^[^ ^44^, ^48^ ^]^ As described in Section S2 (Supporting Information), the predicted relaxation rate associated with actin bending in our composites is $\tau_{b}^{-1}\approx$ 25 s^−1^, which is comparable to the strain rate associated with our fastest speed, $\dot{\gamma }≃\frac{3v}{\sqrt{2}r_{p}}≃$ 23 s^−1^,^[^ ^49^ ^]^ but faster than the two slower rates (≈5.7 s^−1^, ≈11 s^−1^). Thus, we may expect an increased likelihood of stress dissipation on the timescale of the slowest strain rate (v= 6 µm s^−1^) compared to the fastest (v= 24 µm s^−1^). Consistent with this understanding, we find that the viscous drag, which is a measure of stress dissipation within the composite network, is higher at slower speeds, particularly at the highest kinesin concentration (Figure 5E,F).

### 2.3 Hierarchical Structural Heterogeneity Enables Enhanced Mechanical Resistance for Composites

When we consider the mechanical results described above (Figures 3, 4, 5), in the context of the composite restructuring (Figures 1 and 2), we see that the microtubule compaction and network de‐mixing that causes dense small‐scale clustering at intermediate kinesin concentrations also provides mechanical enhancement, as observed by the increase in both stiffness and maximum force. At higher kinesin concentrations the large‐scale phase separation undermines and softens the elastic response. We now aim to quantitatively understand the relationship between de‐mixing and structural heterogeneity and the non‐monotonic dependence of local mechanics on kinesin concentration.

**Figure 6:**
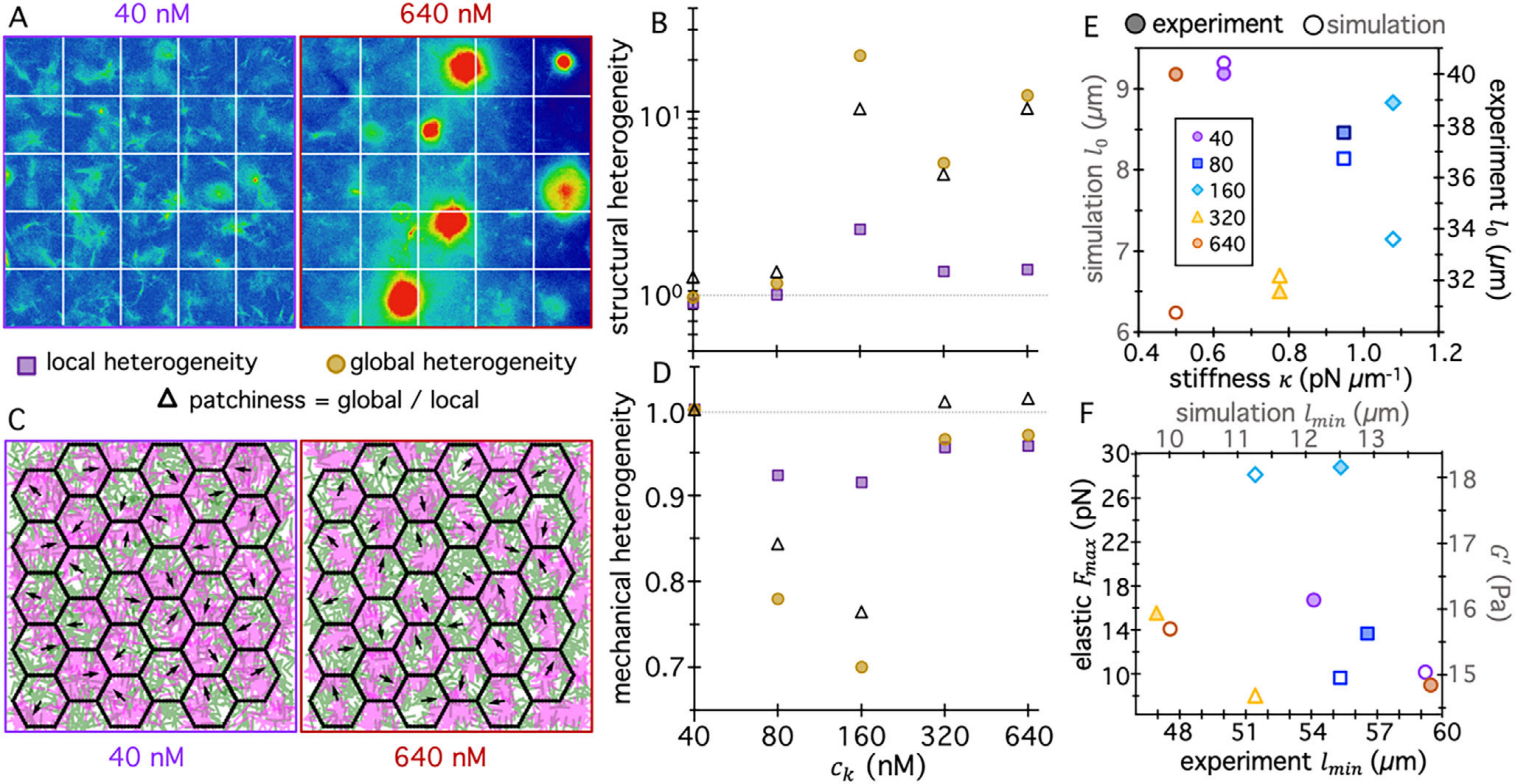
Structural and mechanical heterogeneity and correlations underlying emergent stiffness of active composites. A) Sample epifluorescence images, with pixel intensity values shown in false‐color from low (blue) to high (red), showing labeled actin and microtubules in composites with $c_{k}=$ 40 nm (left, purple border) and $c_{k}=$ 640 nm (right, red border). Each image was divided into a grid of 20 × 20 µm tiles (white lines) to compute local and global heterogeneity and patchiness parameters, $h_{I},H_{I}$ and $p_{I}$. (B) Structural heterogeneity metrics $h_{I}\;$(purple squares), $H_{I}$ (gold circles) and $p_{I}$ (open triangles), normalized by their corresponding $c_{k}=$ 0 nm value, denoted by the dashed horizontal line. C) Sample snapshots of composites snapshots with $c_{k}=$ 40 nm (left, purple border) and $c_{k}=$ 640 nm (right, red border) with overlaid grid of 20 µm hexagonal tiles. The average force exerted on filaments within each tile, depicted as a black arrow, and the standard deviation of force values were used to compute mechanical heterogeneity metrics, $h_{f},H_{f}$ and $p_{f}$, analogous to their structural counterparts. D) Mechanical heterogeneity metrics $h_{f}\;$(purple squares), $H_{f}$ (gold circles) and $p_{f}$ (open triangles), normalized by their corresponding $c_{k}=$ 0 nm value, denoted by the dashed horizontal line. E,F) Correlation plots that display relationships between mechanical ($\kappa ,F_{max},G^{\prime }$) and structural ($l_{0},l_{min}$) parameters measured from experiments (filled symbols, black axis labels) and simulations (open symbols, grey axis labels). All experimental data shown is for 24 µm s^−1^ strains, $F_{max}$ values are for the elastic traces, and simulated $G^{\prime }$ data is for 1 Hz.

Our structural analysis shows varying degrees of clustering over a range of length scales from <10 to ≈100 µm (Figure 2), which encompass the s=20 µm displacement scale used in our optical tweezers experiments, as well as the forced bead displacements in simulations (Figure 3D). To determine the likelihood of observing structural heterogeneity within a local region that a moving particle perturbed, and to better understand the extent of heterogeneity among different regions within an experimental field of view (FOV), we divided the epifluorescence videos we analyzed in Figure 2 into 20 × 20 µm tiles or patches (**Figure** **6**A). For each tile in the FOV, we defined a heterogeneity factor $\delta_{I}=\frac{\sigma_{I}}{I}\;$ from the standard deviation $\sigma_{I}$ and mean I of pixel intensities I. To quantify heterogeneity at local (<s) and global (>s) scales for a given kinesin concentration, we computed the mean and standard deviation of $\delta_{I}$ across all tiles of all videos. The former and latter are measures of local heterogeneity, $h_{I}=\delta_{I}\;$, and global heterogeneity, $H_{I}=\sigma \left(\delta_{I}\right)$, and their ratio $p_{I}=H_{I}\;/h_{I}$ is a measure of structural ‘patchiness’. For reference, for a well‐mixed system, there should be minimal global heterogeneity, i.e., all patches should be identical, so $p_{I}$ should tend to zero. For fractal‐like systems, the heterogeneity would be scale invariant, yielding $p_{I}\approx 1$; and systems that are de‐mixed on the scale of the patches would yield $p_{I}>1$.

False‐color images in Figure 6A depict the extent to which global heterogeneity and patchiness were enhanced and local heterogeneity was suppressed for $c_{k}=$640 nm compared to $c_{k}=$40 nm. We quantified this effect by plotting $h_{I}$, $H_{I}$ and $p_{I}$, normalized by their $c_{k}=$0 values, as functions of $c_{k}$ (Figure 6B). We observed a non‐monotonic dependence on $c_{k}$ with peaks at $c_{k}=$160 nm, consistent with our prior observations. The maximum in $h_{I}$ is a likely indicator of local bundling of filaments that we expect to stiffen the network by stiffening its constituents, consistent with the increased maximum force and stiffness we measured (Figures 3 and 4). The maximum in $H_{I}$ is suggestive of larger scale de‐mixing, and we found similarly high values for $c_{k}=$640 nm, as we expected based on our structural analysis (Figure 2) and visual inspection of the videos (Figure 6A). However, at this highest kinesin concentration, local heterogeneity dropped while the patchiness remained high. Together, these features suggest that larger‐scale aggregation and aster formation, with patches that are largely either filled by a cluster or devoid of microtubules, dominate the force response. This broken connectivity substantially weakens the network; and the prevalence of filament‐poor patches compared to cluster‐spanning patches tips the scales toward a softer mechanical response.

To verify this interpretation and couple structural and mechanical heterogeneity, we again turned to simulations, this time evaluating the distribution of forces exerted on filaments within 20 µm hexagonal tiles (Figure 6C), as fully described in the Supporting Information. We computed similar metrics to assess mechanical heterogeneity, replacing pixel intensity I with force f: $h_{f}=\delta_{f}=\sigma_{f}/f\;\;$ and $H_{f}=\sigma \left(\delta_{f}\right)$, and $p_{f}=H_{f}\;/h_{f}.$ As shown in Figure 6D, in which we plot these metrics normalized by their values for the lowest kinesin concentration (i.e., $c_{k}=$40 nm, metrics are not defined for $c_{k}=$0 nm where f= 0), we observed strong non‐monotonic dependence on $c_{k}$ consistent with our observations of the structural heterogeneity response shown in Figure 6B. However, and notably, all metrics were minimized for the $c_{k}=$160 nm composite, with H and p being most strongly suppressed. This increased homogeneity of forces throughout the composite is consistent with the presence of a well‐connected network of stiff (bundled) fibers that can both efficiently distribute stress and provide strong elastic resistance. At higher $c_{k}$ values, when de‐mixing occurs at larger lengthscales, we observed a much higher patchiness of forces, signifying reduced mechanical connectivity, which is consistent with a weaker force response, loss of stiffness, and enhanced yielding and viscous dissipation.

We have shown clear emergent elasticity of kinesin‐driven composites in both experiments and simulations (Figure 6E,F). This response arises due to de‐mixing of actin and microtubules and is a unique feature of the composite system (Figure S4, Supporting Information). To summarize and provide further insight, we constructed correlation plots to depict the relationships between the structural correlation lengths obtained from SIA of experimental images with mechanical parameters of the network. Specifically, we compared the smaller structural lengthscale $l_{0}$ with stiffness κ (Figure 6E); and the larger lengthscale $l_{min}$ with the experimental maximum force $F_{max}$ and simulated average elastic modulus $G^{\prime }$ (Figure 6F). Across all metrics, we observed maximum elastic response for the $c_{k}=$160 nm composite, as shown by the cyan diamonds being furthest to the right of Figure 6E and the top of Figure 6F. At intermediate stiffness values, we observed good agreement between experimental and simulated dependences of $l_{0}$ on κ, seen as the open and filled symbols closely aligning (Figure 6E). However, at the highest kinesin concentration, $c_{k}=$640 nm, which has the lowest stiffness, simulations report the smallest $l_{0}$ among kinesin concentrations while experiments measured a maximal $l_{0}$. We believe that this distinction (Figure 6E), also seen in Figure 2, can be attributed to the reduced dimensionality and size of the 2D simulations which limits the ability for filaments to move and assemble into large clusters. Despite this simplification, we observe generally similar correlations between mechanical and structural properties for varying kinesin concentrations measured in experiments and simulations (Figure 6F). Comparing $F_{max}$ for the linear traces and the average simulated $G^{\prime }$ values, and their dependences on their respective $l_{min}$ values, we find that simulated and experimental data points at a given kinesin concentration loosely cluster with one another, except for the $c_{k}=$640 nm case for reasons described above. These general features confirm that our simulations are capturing the key physics of the material system, and highlight the importance of coupling between structure and mechanics to produce the emergent behavior.

## 3 Conclusion

We have designed and characterized in vitro composites of actin filaments and microtubules undergoing active restructuring by kinesin motor clusters that pull on microtubules, and found them to transition from interpenetrating networks to de‐mixed microtubule‐rich aggregates and softer actin‐rich phases. Despite this de‐mixing, composites maintain structural integrity, without fracturing or completely phase‐separating, and achieve steady‐states that maintain viscoelastic mechanical properties. We have discovered that this restructuring, seen in both experiments and simulations, leads to rich dependence of the mechanical response on kinesin concentration, including a surprising emergence of enhanced stiffness and elasticity at intermediate kinesin concentrations. This mechanical emergence is coupled to enhanced structural heterogeneity across length scales. Importantly, we previously observed non‐monotonic dependence of mechanical stiffness on passive crosslinking of actin in cytoskeleton composites, suggesting this behavior may be a generalizable feature of crosslinking of one species of a composite. However, in these previous studies, there was no observable de‐mixing, large‐scale bundling, or structural heterogeneity. Rather, the non‐monotonic dependence was a result of modest microscale variations in mesh sizes and fiber stiffnesses. Here, our experiments and simulations demonstrate the importance of hierarchical structural and mechanical heterogeneity in sculpting the mechanical behavior, which we rationalize as a direct result of the internal stress generated by kinesin motors. The distribution of motor‐generated stresses measured in simulations mirrors that of the structural heterogeneity in experiments. Moreover, the stiffening behavior, a unique feature not previously reported in similar passive or active composites,^[^ ^28^, ^35^ ^]^ also may indicate motor‐generated pre‐stress and densification that suppress filament bending and non‐affine deformations that dissipate stress, thereby promoting a stiffening response.

This interplay between structure and mechanics is likely critical to the multifunctionality of the cytoskeleton that allows for wide‐ranging mechanical processes and dynamically sculpts mechanical properties in response to environmental cues and the needs of the cell. Our results and models shed important light on how to engineer and tune composite systems to exhibit emergent mechanics through independent tuning of elastic and viscous contributions of the composite constituents.

## 4 Experimental Section

### Protein Preparation

Rabbit skeletal actin (Cytoskeleton, Inc. AKL99) was reconstituted to 2 mg mL^−1^ in 5 mm Tris‐HCl (pH 8.0), 0.2 mm CaCl_2_, 0.2 mm ATP, 5% (w/v) sucrose, and 1% (w/v) dextran. Porcine brain tubulin (Cytoskeleton, Inc. T240) and HiLyte488‐labeled porcine brain tubulin (Cytoskeleton, Inc. TL488M‐A) were reconstituted to 5 mg mL^−1^ with 80 mm PIPES (pH 6.9), 2 mm MgCl_2_, 0.5 mm EGTA, and 1 mm GTP. All cytoskeleton proteins were flash‐frozen in single‐use aliquots and stored at −80°C.

Biotinylated kinesin‐401^[^ ^21^, ^50^ ^]^ was expressed in Rosetta (DE3) pLysS competent E. coli cells (ThermoFisher), purified, and flash‐frozen into single‐use aliquots, as described previously.^[^ ^33^ ^]^ To prepare force‐generating kinesin clusters, kinesin‐401 dimers were incubated with NeutrAvidin (ThermoFisher) at a 2:1 ratio in PEM‐100 buffer (100 mm PIPES, 2 mm MgCl_2_, 2 mm EGTA) supplemented with 4 µm DTT for 30 min at 4 °C. Clusters were prepared fresh and used within 24 hr.

### Composite Sample Preparation

Composites of actin filaments and microtubules at a 45:55 molar ratio, were polymerized by combining 1.35 µm actin monomers, 1.55 µm tubulin dimers, and 0.1 µm HiLyte488 tubulin dimers in PEM‐100 (100 mm PIPES, 2 mm MgCl_2_, 2 mm EGTA) supplemented with 0.1% Tween, 10 mm ATP, 4 mm GTP, 5 µm Taxol, 1.08 µm phalloidin, and 0.27 µm ActiStain488 phalloidin (Cytoskeleton, Inc. PHDG1‐A) and incubating for 1 hr at 37 °C. The fluorophores for both microtubules and actin were chosen to be spectrally similar to allow both filaments to be visible in a single field‐of‐view with the same excitation/emission filter combination, a requirement due to the fact that the epifluorescence microscope outfitted with the optical tweezers can only accommodate a single fluorescence channel at a time. For optical tweezers experiments, 0.02% (v/v) of 4.5 µm diameter carboxylated microspheres (Polysciences, Inc.), coated with BSA to inhibit non‐specific interactions with the network, were added.^[^ ^28^ ^]^ For two‐color confocal microscopy measurements, HiLyte488 tubulin dimers were replaced with rhodamine tubulin dimers (Cytoskeleton, Inc. TL590M) to allow for separate imaging of actin and microtubules in different channels of the confocal microscope (see below for additional details).

Following polymerization, and immediately prior to experiments, an oxygen scavenging system (45 µg mL^−1^ glucose, 43 µg mL^−1^ glucose oxidase, 7 µg mL^−1^ catalase, 0.005% β‐mercaptoethanol) was added to reduce photobleaching, followed by kinesin clusters at final kinesin concentrations of $c_{k}=$ 0, 40, 80, 160, 320, and 640 nm.

For both optical tweezers and confocal experiments, the final sample was gently flowed into a sample chamber made from a glass slide and coverslip separated by ≈100 µm of double‐sid tape to accommodate ≈10 µL. Both the glass slide and coverslip of the chamber were passivated with BSA prior to flowing in the sample. The chamber was sealed with UV‐curable glue to prevent sample leakage and evaporation. This process completed ≈5 min after the addition of kinesin to the sample, and the sealed sample was incubated for an additional 25 min to allow for motor‐driven restructuring prior to measurements.

The composite mesh size ξ is determined from the mesh sizes for the actin and microtubule networks comprising the composite, $\xi_{A}≃1.46c_{A}^{-1/2}≃$ 1.26 µm and $\xi_{M}≃2.68\;c_{T}^{-1/2}≃$ 2.15 µm, where $c_{A}$ and $c_{T}$ are the molarities of actin and tubulin, via the relation $\xi ≃{\left(\xi_{A}^{3}+\xi_{T}^{3}\right)}^{-1/3}$,^[^ ^44^ ^]^ yielding a composite mesh size of ξ≃ 1.19 µm. The ratio of kinesin clusters to tubulin $R=\frac{c_{k}}{4c_{T}}=$ 0, 0.006, 0.012, 0.024, 0.048, and 0.097 for $c_{k}=$ 0 – 640 nm, where the four accounts for the 4 kinesins (two dimers) per motor cluster.

### Optical Tweezers Microrheology (OTM)

OTM experiments were performed using an optical trap formed by a 1064 nm Nd:YAG fiber laser (Manlight), focused with a 60 × 1.4 NA objective (Olympus), and custom‐built around an Olympus IX71 epifluorescence microscope.^[^ ^41^, ^51^ ^]^ The force was measured using a position‐sensing detector (Pacific Silicon Sensor) to record the deflection of the trapping laser, which over the operating range used within the reported experiments, was proportional to the force acting on the trapped microsphere. The proportionality constant that provides the trap stiffness $k_{OT}$ was determined to be $k_{OT}≃$ 68 pN µm^−1^ using the Stokes drag method.^[^ ^40^, ^41^, ^51^ ^]^ Imaging of the labeled filaments and probes was achieved using a broadband LED source (XCITE) with 488/525 nm excitation/emission filters and a Hamamatsu ORCA‐Flash 4.0LT CMOS camera with a 1024 × 1024 square‐pixel field‐of‐view and frame rate of 20 fps.

For each force measurement, an optically trapped microsphere was dragged back and forth in the ±x‐direction through the sample in a sawtooth pattern using a nanopositioning piezoelectric stage (Mad City Labs) that moves the sample chamber relative to the fixed trap (Figure 3a). The distance the probe was moved in each half‐cycle (i.e., the strain amplitude) and total perturbation time was fixed at s= 20 µm and $t_{f}=$ 50 s for all measurements. The stage position and laser deflection were recorded at 20 kHz, and the stage position was updated at 400 Hz using custom‐written National Instruments LabVIEW programs. Measurements were performed at 3 different speeds for each kinesin concentration:v = 6, 12, 24 µm s^−1^. Prior to each strain and force measurement, an image of the labeled filaments surrounding the probe in a 202 × 135 µm (900 × 600 pixel) area centered at the center of the strain path was captured. The same protocol was repeated but using a piezoelectric mirror to move the trapped bead relative to the sample (keeping the sample chamber fixed), and recording 1000‐frame videos of the labeled filaments. These videos were used in the image analysis presented in Figures 2 and 6.

For each ($v,c_{k}$) combination, measurements were repeated using multiple beads located in different regions of the sample chamber, which were separated by ≥100 µm. This suite of measurements for each condition was then repeated for three different samples for a total of ≥24 beads per condition. While cyclic straining was performed for each measurement, in which the same probe was repeatedly pulled through the material, a significant number of probes were lost on the return after the initial pull, and for those that were not lost, the measured force ranges for subsequent pulls were considerably smaller, suggesting that the initial pull caused network damage or plastic deformation. Thus, data presented in Figures 3, 4, 5, 6 are solely for the initial loading cycle.

Post‐acquisition analysis of measured force F(x,t) (Figures 3, 4, 5, 6) was performed using custom‐written MATLAB scripts. Each 50 s measurement was divided into individual cycles and the second half of each cycle, when the probe was moving in the −x direction, was removed. The last 1% of the forward cycle (0.2 µm) was also removed to avoid artifacts that arise from attempting to instantaneously switch the stage motion from +v to −v (the stage response rate was 400 Hz). Average data shown for each condition represent averages over all valid trials and error bars represent standard error. Trials were considered invalid if the bead was pulled out of the trap.

To characterize the composite structure, Spatial Image Autocorrelation (SIA) analysis^[^ ^39^, ^51^ ^]^ was performed on each frame of each of the 1000‐frame videos described above. SIA measures the correlation in intensity $g_{I}$ of two pixels in an image as a function of separation distance r. Autocorrelation curves $g_{I}\left(r\right)$ were generated by taking the fast Fourier transform of the image F(I), multiplying by its complex conjugate, applying an inverse Fourier transform $F^{-1},$ and normalizing by the squared intensity: $g_{I}\;\left(r\right)=\frac{F^{-1}\left({\left|F(I(r))\right|}^{2}\right)}{{\left[I(r)\right]}^{2}}\;$. To determine the effect of motor activity on the structure, $g_{I}\left(r\right)$ for the no‐motor case ($c_{k}=$0) was subtracted from $g_{I}\left(r\right)$ for each kinesin concentration: $\Delta g_{I}\;\left(r,c_{k}\right)=g_{I}\left(r,c_{k}\right)-g_{I}\left(r,0\right)$, which was shown in Figure 2. No dependence on strain speed was observed, so the data shown was the average and standard error across all frames of all videos for a given kinesin concentration $c_{k}$.

### Fluorescence Confocal Microscopy

To determine the unperturbed composite structure and dynamics, videos of composites with distinctly‐labeled actin and microtubules were recorded using a Nikon A1R laser scanning confocal microscope with a 60 × 1.4 NA oil‐immersion objective (Nikon), 488 nm laser with 488/525 nm excitation/emission filters (to excite/image actin), and 561 nm laser with 565/591 nm excitation/emission filters (to excite/image microtubules) (Figure 1). For each kinesin concentration, four time‐series (videos) of 512 × 512 square‐pixel (213 × 213 µm) images were collected at 1.86 fps for a total of 1116 frames (10 min). Videos were collected at 10, 20, 30, and 40 min after the addition of kinesin motors, with each video collected in a different field of view separated by >500 µm. No dependence of the composite structure on the time that the video was acquired was observed, indicating the motor activity was largely halted after 10 min. All videos include two channels that separate the actin and microtubule signals such that they can be processed separately and compared.

### Computational Model

A simple Lattice‐gas model of microfilament composites was recently developed, which adequately captures experimentally observable filament restructuring in the composite (see Ref. [33]). Here, this model was further developed to predict the restructuring of the composites, and the resulting changes in composite mechanics due to varying levels of motor activity. In this model, the available space was defined as a hexagonal grid with periodic boundary conditions. Each grid point can be occupied by a single microtubule or single actin filament center or can be empty. The filaments can interact with neighbors within reach, via 1) motor‐generated forces that can either pull the interacting filaments toward each other or push them away from each other; and 2) motor crosslinks that increase the friction forces on the interacting filaments and allow forces to be transmitted through crosslinked filament clusters. The movement of a filament center to a neighboring grid point within a small temporal time step is then a stochastic event whose probability can be calculated using the transition rate based on the first passage times.

A minimal approach to capture the dynamics was purposely chosen to shed light on the competing factors of activity and friction. The model assumes a single length for all filaments of l=5 µm while in experiments actin and microtubules assume distributions of lengths ($l_{A}≃4\pm 3$ µm and $l_{M}≃8\pm 4$ µm).^[^ ^44^ ^]^ All filaments are treated as rigid rods, while actin filaments in experiments are semiflexible with a persistence length of ≈17 µm. The model also assumes uniform motor density throughout the simulation space, which might locally under or overestimate motor‐generated forces within the composites. Finally, the simulations are in 2D while experimental composites span 3D.

The model was implemented on a 100 × 100 µm 2D space with a hexagonal lattice, where the lattice spacing is 1.25 µm. Each 5‐µm long filament interacts with other filaments located within four grid points in all directions. Initially, each lattice point is either occupied by a single microtubule center, a single actin filament center, or is left empty using probabilities matching the average volume fraction occupied by these elements. The initial orientations of microtubules and actin filaments were randomly distributed. Single occupancy per lattice site was enforced for filament centers, but the extended length of each filament allows for interactions with other filaments within its interaction radius. At any snapshot in time, each filament has a specific orientation that is a function of the net force on the filament due to motor forces between interacting filaments.

The movement of the filaments was simulated in each iteration by calculating the likelihood of each possible movement, $p_{ij}$ for all grid points i and j, where at least one of them contains a filament center, and randomly picking one of these movements to occur based on these probabilities. The simulation was run for $T_{S}=5$ min, which was found to be sufficient to reach quasi‐steady state. The model calculations and simulations are coded in python and the scripts are available on GitHUB.^[^ ^52^ ^]^ A cartoon depiction of the model is shown in Figure 1 and Figure S1 (Supporting Information) and numerical values for all model parameters are included in Table S1 (Supporting Information).

To quantify the degree of clustering and segregation of the different filaments, The filament pair distribution function $g_{AM}\left(r\;,T\;\right)$, where the subscripts A and M represent the filament type (actin or microtubules), quantifies the degree of clustering and segregation of the different filaments by giving the probability of finding a filament (actin or microtubule) a radial distance r from any other like filament:

(2) $$g_{AA}\;\left(r\right)=\langle \frac{N_{A}\left(r\right)}{f_{A}N\left(r\right)}\rangle ,g_{MM}\;\left(r\right)=\langle \;\frac{N_{M}\left(r\right)}{f_{M}N\left(r\right)}\rangle$$

or unlike filament

(3) $$g_{MA}\;\left(r\right)=\langle \frac{N_{A}\left(r\right)}{f_{A}N\left(r\right)}\rangle ,g_{AM}\;\left(r\right)=\langle \frac{N_{M}\left(r\right)}{f_{M}N\left(r\right)}\rangle$$

where $N_{A}\left(r\right)$ is the number of neighboring filaments of type A a distance r from a specific filament, $f_{A}$ is the volume fraction of filament A in the simulation space, and N(r) is the maximum number of possible neighbors a distance r from the specific filament. An increase in $g_{AA}\left(r\right)$ above 1 indicates clustering of like filaments, and a decrease in $g_{MA}\left(r\right)$ below 1 indicates segregation of unlike filaments. As with experimental SIA data, the distribution for the no‐motor case is subtracted from that for each kinesin concentration to yield: $\Delta g_{AA}\;\left(r,c_{k}\right)=g_{AA}\left(r,c_{k}\right)-g_{AA}\left(r,0\right),$ which is plotted in Figure 2a–c. Correlation analysis was performed up to r= 25 µm which was found to be sufficient to capture most of the correlation decay.

The storage modulus $G^{\prime }$ and loss modulus $G^{\prime \prime }$ of each quasi‐steady state composite were calculated by embedding a spherical bead of radius $r_{sph}=$ 0.625 µm into the in silico composite and applying a sinusoidal force on the bead with amplitude $F_{0}=$ 100 pN and oscillation frequencies ω= 0.25, 0.5, 1 Hz for 20 full periods. The resulting displacement of the bead through the composite was measured, and the magnitude x and phase angle $\varphi_{x}$ of the displacement at the chosen forcing frequency ω was computed using fast Fourier transform analysis By combining this data with the known amplitude $F_{0}\;$ and phase angle $\varphi_{F}$ of the applied force, the viscoelastic moduli were calculated as

(4) $$G^{\prime }=\frac{F_{0}}{x}\;*\cos\phi *\frac{1}{r_{sph}}$$

(5) $$G^{\prime \prime }=\frac{F_{0}}{x}\;*\sin\phi *\frac{1}{r_{sph}}$$

where $\phi =\varphi_{F}\;-\varphi_{x}$ is the phase difference. The relative elasticity of the response was quantified by evaluating the inverse loss tangent ${\left[\tan\phi \right]}^{-1}=G^{\prime }\;/G^{\prime \prime }$ which was greater or less than 1 for elastic‐dominated and viscous‐dominated responses, respectively. Each data point shown in Figure 3 and Figures S2 and S3 (Supporting Information) was the average over ten beads in each of the three replicate samples and normalized by the corresponding value for $c_{k}=$ 0. Error bars were computed by bootstrapping of ten random subsets of the data as described in Supporting Information.

## Conflict of Interest

The authors declare no conflict of interest.

## Author Contributions

J.S. and R.M.R.A. conceived and designed the research. J.S., C.G., K.M., M.S., P.K., M.T.V., and R.M.R.A. performed research and analyzed data. R.M.R.A., C.G., P.K., and M.T.V. prepared figures and wrote the manuscript. P.K. and R.M.R.A. supervised the research. J.L.R. prepared reagents. All authors interpreted data and edited the manuscript.

## Supporting information



Supporting Information

## Data Availability

The data that support the findings of this study are available from the corresponding author upon reasonable request.
