# Supplementary material for: Kinesin‐Driven De‐Mixing of Cytoskeleton Composites Drives Emergent Mechanical Properties
Source: Macromol Rapid Commun. 2025 Apr 10;46(14):2401128. doi: 10.1002/marc.202401128 (PMC12272537; doi:10.1002/marc.202401128)
Supplement: Supplementary file 1 — Supporting Information [file MARC-46-2401128-s001.pdf]

Macromolecular Rapid Communications

Supporting Information

for *Macromol. Rapid Commun.*, DOI 10.1002/marc.202401128

Kinesin-Driven De-Mixing of Cytoskeleton Composites Drives Emergent Mechanical Properties

*Janet Sheung, Christopher Gunter, Katarina Matic, Mehrzad Sasanpour, Jennifer L. Ross, Parag Katira, Megan T. Valentine and Rae M. Robertson-Anderson**

Kinesin-driven de-mixing of cytoskeleton composites drives emergent mechanical properties

Janet Sheung[1], Christopher Gunter[2], Katarina Matic[3], Mehrzad Sasanpour[3], Jennifer L. Ross[4], Parag Katira[2], Megan T. Valentine[5], Rae M. Robertson-Anderson[3*]

[1]Department of Natural Sciences, Scripps and Pitzer Colleges, Claremont, CA, United States; W. M. Keck Science Department, Claremont Mc College, Claremont, CA 91711, USA
[2]Department of Mechanical Engineering, San Diego State University, San Diego, CA 92182, USA
[3]Department of Physics and Biophysics, University of San Diego, San Diego, CA 92110, USA
[4]Department of Physics, Syracuse University, Syracuse, NY 13244, USA
[5]Department of Mechanical Engineering, University of California, Santa Barbara, Santa Barbara CA 93106, USA

*Corresponding Author: randerson@sandiego.edu

Supplemental Information

Section S1. Computational Model

Table S1: Parameters used in mathematical model and simulations

Figure S1: Sample plot showing simulation mechanics

Section S2. Predicted Relaxation Timescales

Section S3. Mechanical Circuit Model

Figure S2. Average force traces classified by response type for strains of speed $v = 6$ µm s^{-1} (top) and $v = 12$ µm s^{-1} (bottom).

Figure S3. Viscoelastic moduli determined from simulated oscillatory forcing of a bead through active cytoskeletal composites at varying oscillation frequencies.

Figure S4. Viscoelastic moduli determined from simulated oscillatory forcing of a bead through an active microtubule network without actin present.

Figure S5. Mechanical circuit model captures the viscoelastic behaviour of elastic and yielding response classes.

Figure S6. Ensemble of force traces comprising averages shown in Figure 4

Supplementary References

Section S1.

Computational Model: We have recently developed a simple Lattice-gas model of microfilament composites which adequately captures experimentally observable filament restructuring in the composite[1]. We build on this model to predict the restructuring of the composites, and the resulting changes in composite mechanics due to varying levels of motor activity. In this model, the available space is defined as a hexagonal grid with periodic boundary conditions. Within the model, each grid point can be occupied by either a single actin filament or single microtubule center or can be empty. The initial orientations of microtubules and actin filaments are random distributed. There is a single occupancy per lattice site rule for filament centers, but the extended length of each filament allows for interactions with other filaments within its interaction radius. The filaments can interact with neighboring filaments within reach, via 1) motor-generated forces that can either pull the interacting filaments towards each other or push them away from each other; and 2) crosslinks that increase the friction forces on the interacting filaments and allow forces to be transmitted through crosslinked filament clusters. We implemented a parameter that designates a portion of the motors as active, in which case they exert forces on interacting filaments, and a portion as passive, in which case they crosslink interacting filaments together without exerting force. The movement of a filament center to a neighboring grid point within a small temporal time step is then a stochastic event whose probability can be calculated using the transition rate based on the first passage time. This probability is given by

$$p_{ij} = \frac{k_{ij}}{\sum k_{ij}} \quad \text{S1,}$$

where k_{ij} is the rate constant describing the transition rate of a filament at grid location i in the direction of grid location j. This rate constant is calculated as

$$k_{ij} = \frac{k_0}{f_i} + \frac{v_i \cdot \delta}{s} \quad \text{S2,}$$

Where k_0 is the free filament transition rate, f_i is the friction factor associated with the filament, v_i is the velocity of the filament, δ is a unit vector in the direction of motion from grid location i to grid location j, and s is the distance between the two grid locations.

The friction factor of a single filament is given by

$$f_i = 1 + \frac{\gamma_{mot} * (N_{mot,act} + N_{mot,pas}) * N_{fil}}{\gamma_{fil}} \quad \text{S3,}$$

where γ_{mot} is the friction coefficient of a single motor protein (γ_k for a kinesin motor), $N_{mot,act}$ and $N_{mot,pas}$ are the number of active and passive motor proteins per filament (randomly selected from a Poisson distribution based on the mean value), N_{fil} is the number of filaments within an interaction distance, and γ_{fil} is the friction coefficient of a single filament (γ_M for a microtubule).

The velocity term is given by

$$v_i \cdot \delta = \frac{F_i \cdot \delta}{\gamma_{fil} + \gamma_{mot} * (N_{mot,act} + N_{mot,pas}) * N_{fil}} \quad \text{S4,}$$

where F_i is the net force generated by the motors between filament i and all same-type filaments within an interaction distance. This net force is given by

$$F_i = \sum F_{ij} \quad \text{S5,}$$

where F_{ij} is given by the force per motor (F_k for kinesin) times the number of active motors per filament ($N_{k,act}$), which is randomly selected from a Poisson distribution based on the mean value. The direction of F_{ij} is along the line joining the two filament centers and can be attractive

or repulsive. This results in a filament orientation which is aligned with the direction of the vector sum of the forces exerted by all filaments of the same type within an interaction distance.

The movement of a filament center to a neighboring grid point occupied by another filament center is restricted sterically and can be only accomplished if the two filaments exchange positions. Thus, in such a scenario, the movement probability of filament i to a neighboring grid point containing filament j's center is given by

$$p_{i\leftrightarrow j} = p_{j\leftrightarrow i} = p_{i\to j} \times p_{j\to i} \quad \text{S6,}$$

which is the same for the filament at grid point j exchanging its location with filament at i.

In the same spirit, the movement of a filament from grid point i to a neighboring empty grid point j is given by

$$p_{i\leftrightarrow j} = p_{i\to j} \times 1 \quad \text{S7.}$$

We purposefully chose a minimal approach to capture the composite dynamics to shed light on the competing factors of motor activity and friction from crosslinkers. Within this simplified approach, out model assumed a single length for all filaments, while in experiments actin and microtubules display a distribution of lengths. We treated all filaments as rigid rods while actin in experiments is semiflexible with a persistence length of ~17 µm. Our simulations were executed in 2D while experimental composites span 3D space.

We implemented our model on a 100 µm x 100 µm 2D space with a hexagonal lattice, with a lattice spacing of 1.25 µm. Each microtubule or actin filament was assumed to be 5 µm in length, such that each filament interacted with other filaments located within 4 grid points in all directions. Initially, each lattice point was either occupied with a microtubule center, an actin filament center, or was left empty using probabilities matching the average volume fraction occupied by these elements. The movement of the filaments was simulated in each iteration by calculating the likelihood of each possible movement, p_{ij} for all grid points i and j, where at least one of them contains a filament center, and randomly picking one of these movements to occur based on these probabilities. Since each movement occurred over a timescale of $\frac{1}{k_{ij}}$, the effective time progression for a single movement to have occurred was approximated by selecting a random value from an exponential distribution with a mean of $\frac{1}{\sum k_{ij}}$ ($i \neq j$, and at least i or j was occupied by a filament center) at each iteration step, following the Gillespie algorithm. Thus, Δt, the time progression, at each iteration step was dynamically adjusted to match the ongoing system dynamics. The simulation was run for $T_S = 5$ minutes, which we find to be sufficient to reach quasi-steady state. Specifically, running the simulation for $0.5T_S$, $0.8T_S$, T_S, and $1.2T_S$ iterations, we observed insignificant change in the filament distributions for $\geq 0.8T_S$. Additionally, we observed that the sum of all rate constants for each filament type reaches a quasi-stable non-zero value, implying quasi-steady state kinetics. The model calculations and simulations were coded in Python and the scripts are available on GitHUB (https://github.com/compactmatterlab/active-filament-networks/2024)[2]. A schematic depiction of the model is shown in Fig S1 and numerical values for all model parameters are included in Table S1.

Structural analysis: To quantify the degree of clustering and segregation of the different filaments, we computed the filament pair distribution function $g_{ij}(r, T)$ where the subscripts i

and j represent the filament type, either actin (A) or microtubules (M), and which gives the probability of finding a filament (actin or microtubule) a radial distance r from any other filament. The probability of finding a filament (actin or microtubule) a radial distance r from any other like filament is given by:

$$g_{AA}(r) = \langle \frac{N_A(r)}{f_A N(r)} \rangle, g_{MM}(r) = \langle \frac{N_M(r)}{f_M N(r)} \rangle \qquad \text{S8,}$$

or unlike filament

$$g_{MA}(r) = \langle \frac{N_A(r)}{f_A N(r)} \rangle, g_{AM}(r) = \langle \frac{N_M(r)}{f_M N(r)} \rangle \qquad \text{S9,}$$

where $N_A(r)$ is the number of neighboring filaments of type A a distance r from a specific filament, f_A is the volume fraction of filament A in the simulation space, and $N(r)$ is the maximum number of possible neighbors a distance r from the specific filament. An increase in $g_{AA}(r)$ above 1 indicates clustering of like filaments, and a decrease in $g_{MA}(r)$ below 1 indicates segregation of unlike filaments. As with experimental SIA data, we subtracted the distribution for the no-motor case from that obtained for each kinesin concentration to yield: $\Delta g_{AA}(r, c_k) = g_{AA}(r, c_k) - g_{AA}(r, 0)$, which we plotted in Fig 2a-c. We performed correlation analysis up to $r =$ 25 μm which we found sufficient to capture most of the correlation decay. Each scenario was simulated for 10 iterations and the results were combined to determine the average value and standard error.

Viscoelastic response: We calculated the storage modulus G' and loss modulus G'' of each quasi-steady state composite by embedding a spherical bead of radius $r_{sph} = 0.625$ μm into the *in-silico* composite and applying a sinusoidal force on the bead with amplitude $F_0 = 100$ pN and oscillation frequencies $\omega =$ 0.25, 0.5, 1 Hz for 20 full periods. We measured the resulting displacement of the bead through the composite, and used fast Fourier transform analysis to compute the magnitude x and phase angle φ_x of the displacement at the chosen forcing frequency ω. By combining this data with the known amplitude F_0 and phase angle φ_F of the applied force, we calculated the viscoelastic moduli as

$$G' = \frac{F_0}{x} * \cos\phi * \frac{1}{r_{sph}} \qquad \text{S10,}$$

$$G'' = \frac{F_0}{x} * \sin\phi * \frac{1}{r_{sph}} \qquad \text{S11,}$$

where $\phi = \varphi_F - \varphi_x$ is the phase difference. We quantified the relative elasticity of the response by evaluating the inverse loss tangent $[\tan\phi]^{-1} = G'/G''$ which is greater or less than 1 for elastic-dominated and viscous-dominated responses, respectively. Each data point shown in Figs 3, S3,S4 represented an average over 10 beads in each of 3 replicate samples.

We performed this analysis at 3 different frequencies (0.25 Hz, 0.5 Hz, and 1 Hz) and applied the sinusoidal force to the spherical bead for 20 full periods. Each scenario was simulated for 30 iterations and a bootstrapping technique was used to determine the average value and standard error. In this technique, a sample of 10 iterations is randomly selected from the pool of 30 iterations, and the results are combined to determine an average value for that sample. This process was performed 10 times, and the results from the 10 samples were then used to determine the final average value and standard error.

Force heterogeneity: We calculated the local and global heterogeneity of the forces exerted on filaments throughout each composite by first superimposing a grid of 30 hexagonal tiles, each with a long diagonal of 20 μm, over the composite network. For each tile, we evaluated the net force f acting at each grid location within the tile, and computed the mean $\langle f \rangle$ and standard

deviation σ_f of the force ensemble, from which we determined the mechanical heterogeneity within each tile $\delta_f = \frac{\sigma_f}{\langle f \rangle}$. We computed the local and global mechanical heterogeneity factors, h_f and H_f, as the mean and standard deviation of the 30 individual δ_f values: $h_f = \langle \delta_I \rangle$ and $H_I = \sigma(\delta_I)$.

	**Description**	**Value**	**Reference**
Total Grid Size		100 μm x 100 μm	
% actin filaments	% of 2D space taken up by actin filament	25%	experimental
% microtubules	% of 2D space taken up by microtubules	30%	experimental
Grid spacing (l)	Distance between grid locations	1.25 μm	
Filament length	Length of each actin filament and microtubule	5 μm	experimental
F_k	Force generated per kinesin motor	6 pN	[3]
$N_{k,act}$	Number of active kinesin motors per microtubule-microtubule interaction	variable	experimental
$N_{k,pas}$	Number of passive kinesin motors per microtubule-microtubule interaction	variable	experimental
γ_A	Viscous drag on an actin filament	0.005 pN*ms/nm	[4]
γ_M	Viscous drag on a microtubule filament	0.01 pN*ms/nm	[5]
γ_k	Viscous drag on the filament due to single kinesin motor binding	6 pN*ms/nm	[5]
$k_{0,A}$	Free filament transition rate for actin filament	1.28 s^{-1}	calculated using first passage time to a neighboring grid point
$k_{0,M}$	Free filament transition rate for microtubule	0.64 s^{-1}	Calculated using first passage time to a neighboring grid point
F_0	Force applied to spherical bead	100 pN	experimental
r_{sph}	Radius of spherical bead	0.625 μm	lattice resolution

Table S1: Parameters used in mathematical model and simulations. Specific numerical values of parameters were chosen to match experimental conditions, including the concentrations of actin, microtubules, and kinesin. Values for motor forces and viscous drag terms were based on literature values, as specified.

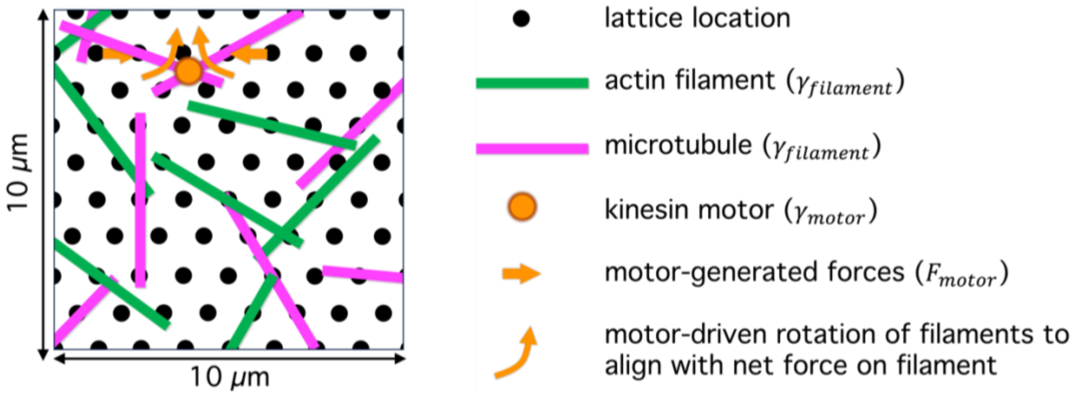

Figure S1: Sample plot showing simulation mechanics. The actin filaments and microtubules exist on a lattice of grid points. There is a drag ($\gamma_{filament}$) associated with their movement. Motor proteins exert forces which drive movement of the filaments (F_{motor}) but also exert drag (γ_{motor}).

Section 2. Predicted Relaxation Timescales

Several predicted relaxation timescales corresponding to various mechanisms have been shown to play a role in the force response of cytoskeleton networks[6]. The shortest relaxation timescale is that over which hydrodynamic interactions (HI) between filaments become important, termed the mesh time $\tau_\xi \approx \epsilon\xi^4 l_p^{-1}$ where ξ is the mesh size, l_p the persistence length and $\epsilon = \zeta/k_B T$ is the friction term, which we assume to be of order ~1 s μm^{-3}.[7,8] This expression yields $\tau_{\xi,A} \approx$ 118 ms and $\tau_{\xi,M} \approx$ 2 ms for the actin filaments and microtubules in the composites which equate to relaxation rates of $\tau_{\xi,A}^{-1} \approx$ 8.5 s^{-1} and $\tau_{\xi,M}^{-1} \approx$ 500 s^{-1}. We can compare these values to our experimental strain rates, $\dot{\gamma} \simeq 3v/\sqrt{2}r_p \simeq$ 5.7, 11.3, 22.6 s^{-1}, where v and r_p are the speed and radius of the optically trapped probe[9]. The fact that the mesh rates for both filaments are faster than or similar to all strain rates suggest that HI are important across the entire range of strains. In other words, because $\dot{\gamma} \lesssim \tau_\xi^{-1}$ the filaments have enough time to interact through HI over the timescale over which the strain is applied. In cases in which $\dot{\gamma} > \tau_\xi^{-1}$, the response is expected to be that of single non-interacting filaments. Another important timescale for semiflexible actin is the timescale associated with bending $\tau_b \approx [4\pi\eta_s/l_p k_B T\, ln\,(2\xi/d)](2L/3\pi)^4$ where d and L are the filament diameter and length and η_s is the solvent viscosity. A representative average actin filament length of $L \approx$ 5 μm yields $\tau_b \approx$ 39 ms and $\tau_b^{-1} \approx$ 25 s^{-1}, a rate that is comparable to or faster than all measured strain rates. This relation suggests that actin filaments are able to bend in response to the applied strains to dissipate stress, rendering bending modes to be likely contributors to the composite response.

Section 3. Mechanical Circuit

To capture the dynamic force-response of the composite network to local deformation via the motion of an optically-trapped particle, we constructed a mechano-equivalent circuit model as follows:

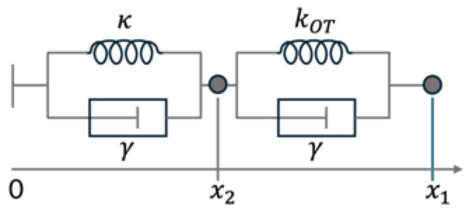

where x_1 denotes the position of the center of mass position of the optical trap and x_2 denotes the center of mass position of the particle. Within the model, we assume

$$\frac{dx_1}{dt} = v \quad \text{S12}$$

$$x_1 = vt + L_0 \quad \text{S13}$$

where L_0 is $x_1 \ at \ t = 0$. Note that this construction assumes a different frame of reference than in the experimental case, in which the optical trap, rather than the stage, is displaced at a fixed speed v. Thus, forces within the model are calculated as a function of x_1, whereas the experimental data are plotted as force versus stage displacement x . We treat these two independent parameters as equivalent. By balancing the forces in the two Kelvin-Voigt modules, we found

$$k_{trap}(x_1 - x_2) + \gamma \frac{d}{dt}(x_1 - x_2) = \kappa(x_2) + \gamma \frac{d}{dt}(x_2) \quad \text{S14}$$

which simplifies to

$$\frac{dx_2}{dt} + \frac{(\kappa + k_{trap})}{2\gamma} x_2 = \frac{k_{trap} v}{2\gamma} t + \frac{(k_{trap} L_0 + \gamma v)}{2\gamma} \quad \text{S15}$$

Solving for $x_2$under the assumption that at $t = 0, x_2 = x_1 = L_0 \sim 0$ we get

$$x_2 = \frac{k_{trap}}{k_{trap} + \kappa} vt + \frac{\gamma v}{k_{trap} + \kappa} (1 - \frac{2k_{trap}}{k_{trap} + \kappa})(1 - e^{-\left(\frac{k_{trap} + \kappa}{2\gamma}\right)t}) \quad \text{S16}$$

To find the force as a function of trap displacement, x_1 we asserted $F(x_1) = k_{trap}(x_1 - x_2)$. Substituting $t = \frac{x_1}{v}$ in Eqn. S16 above, we obtain

$$F(x_1) = \frac{k_{trap}\kappa}{k_{trap} + \kappa} \cdot x_1 - \gamma v k_{trap} \left(\frac{\kappa - k_{trap}}{\left(k_{trap} + \kappa\right)^2}\right)\left(1 - e^{-\left(\frac{k_{trap} + \kappa}{2\gamma}\right)x_1}\right)$$

which provides a predictive relationship between stage displacement, speed, and force.

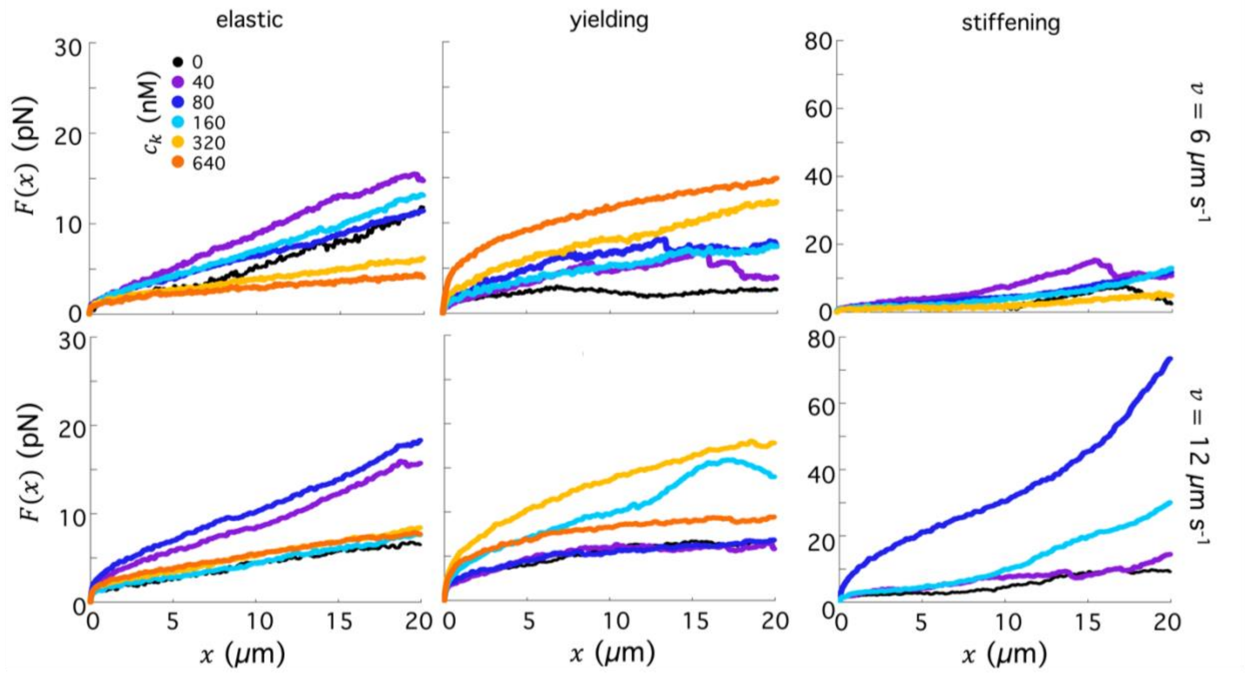

Figure S2. Average force traces classified by response type for strains of speed $v = 6$ μm s^{-1} (top) and $v = 12$ μm s^{-1} (bottom). Average force $F(x)$ versus stage position x for each kinesin concentration c_k, listed and color-coded according to the legend in the top left panel. Force traces classified as elastic (left), yielding (middle), and stiffening (right) are averaged separately. The highest kinesin concentrations lacked stiffening traces at some speeds, in which case no data are shown in the righthand panel. The data for $v = 24$ μm s^{-1} are shown in Figure 4A.

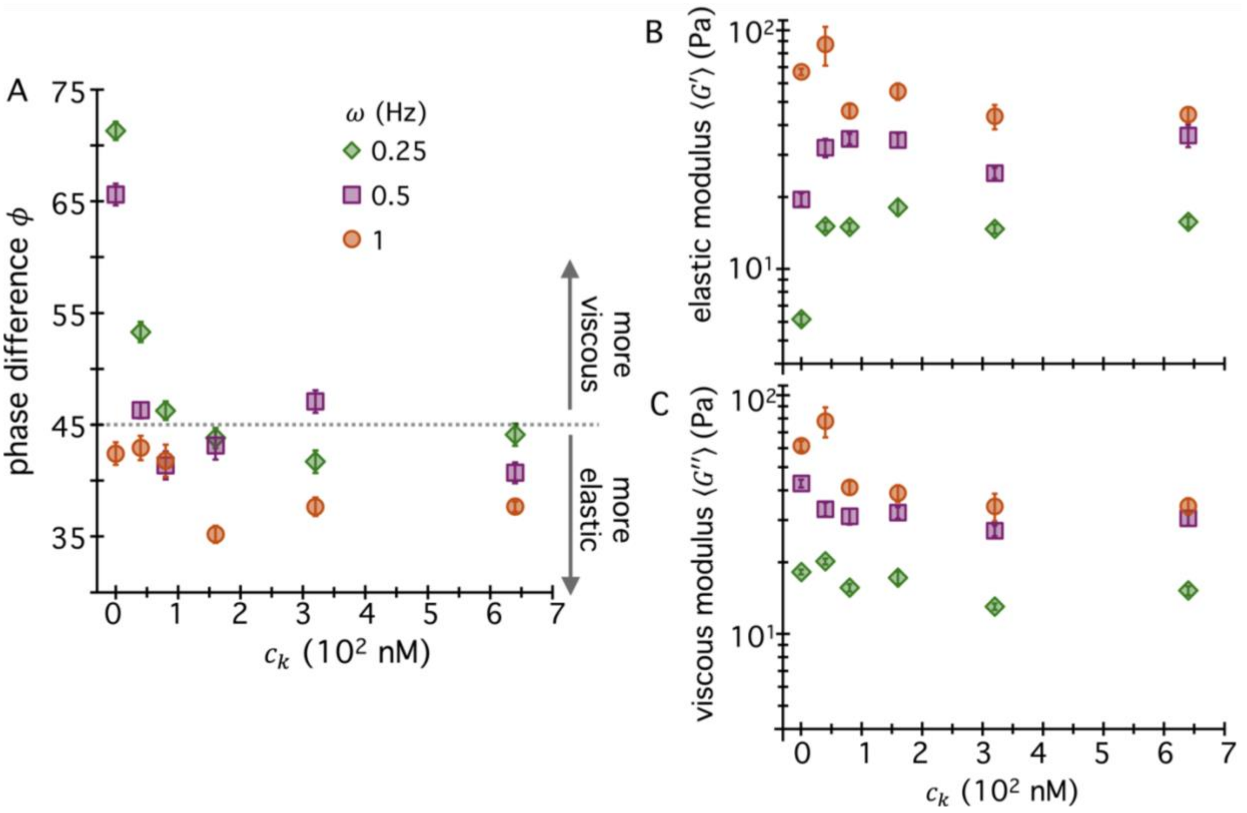

Figure S3. Viscoelastic moduli determined from simulated oscillatory forcing of a bead through 2D active cytoskeletal composites at varying oscillation frequencies. (A) The phase difference ϕ (in units of degrees) between the force oscillation and bead displacement oscillation as a function of kinesin concentration c_k. Force oscillations were performed at frequencies of $\omega =$ 0.25 (green diamonds), 0.5 (purple squares) and 1 (red circles) Hz. $\phi = 0$ and $\phi = 90$ correspond to purely elastic and viscous behaviour, respectively, as indicated by the arrows. The dashed line at $\phi = 45°$ indicates equal viscous and elastic contributions. (B,C) The elastic and viscous moduli, G' (B) and G'' (C), computed from the phase difference, force F, and bead displacement x as described in SI Section 1.

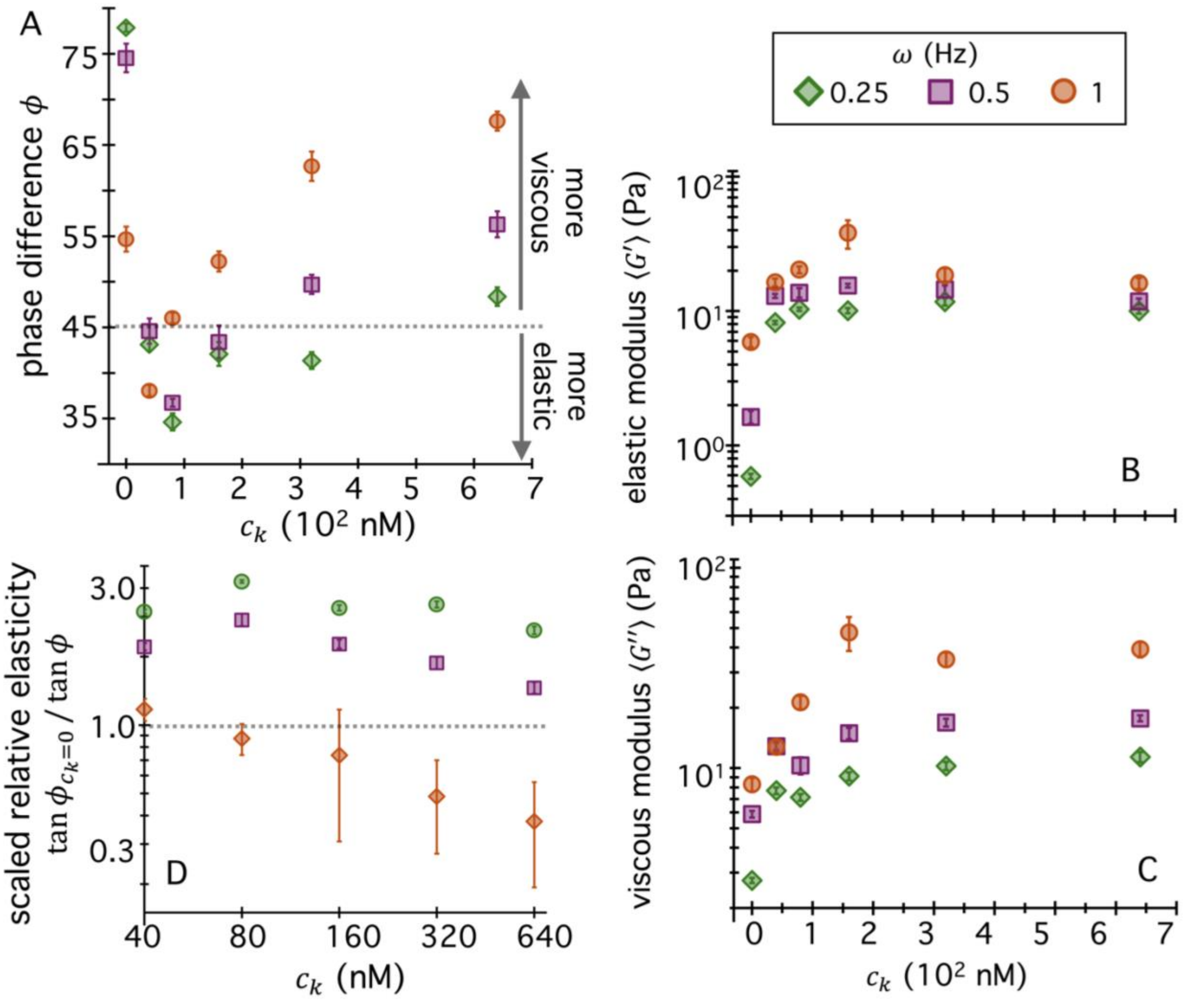

Figure S4. Viscoelastic moduli determined from simulated oscillatory forcing of a bead through active microtubule networks (no actin) at varying oscillation frequencies. Same simulations and data as presented in Figure 4C-E and S4, but without actin. All other concentrations and parameters are the same. (A) The phase difference ϕ (in units of degrees) between the force oscillation and bead displacement oscillation as a function of kinesin concentration c_k. Force oscillations were performed at frequencies of $\omega = 0.25$ (green diamonds), 0.5 (purple squares) and 1 (red circles) Hz. $\phi = 0$ and $\phi = 90$ correspond to purely elastic and viscous behavior, respectively, as indicated by the arrows. The dashed line at $\phi = 45^{\circ}$ indicates equal viscous and elastic contributions. (B,C) The elastic and viscous moduli, G' (B) and G'' (C), computed from the phase difference, force F, and bead displacement x as described in SI Section 1. (D) Scaled relative elasticity, computed as the inverse loss tangent $[\tan \phi]^{-1}$ normalized by the corresponding $c_k = 0$ value, indicated by the dashed horizontal line, versus kinesin concentration. Color and symbol scheme is the same for all panels and shown above panel B. Error bars in all panels correspond to standard deviation of bootstrapped ensembles.

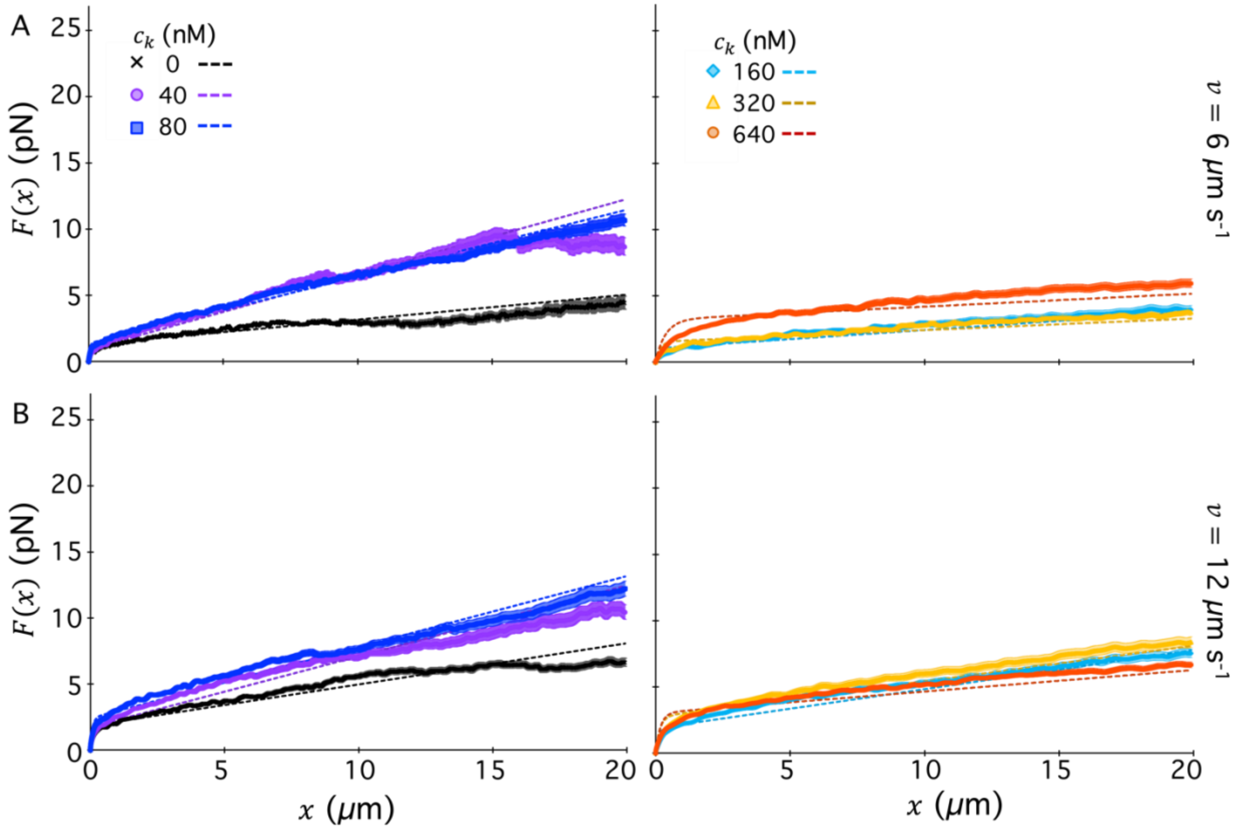

Figure S5. Mechanical circuit model captures the viscoelastic behavior of elastic and yielding response classes. Force $F(x)$ versus stage position x, averaged across all elastic and yielding traces for each kinesin concentration c_k, listed and color-coded according to the legend, for (A) v = 6 μm s^{-1} and (B) v = 12 μm s^{-1}. Error bars denote standard error of the mean. Dashed lines are fits to the equation of motion for the mechanical circuit depicted in Fig 5A. Data for v = 24 μm s^{-1} is shown in Fig 5B.

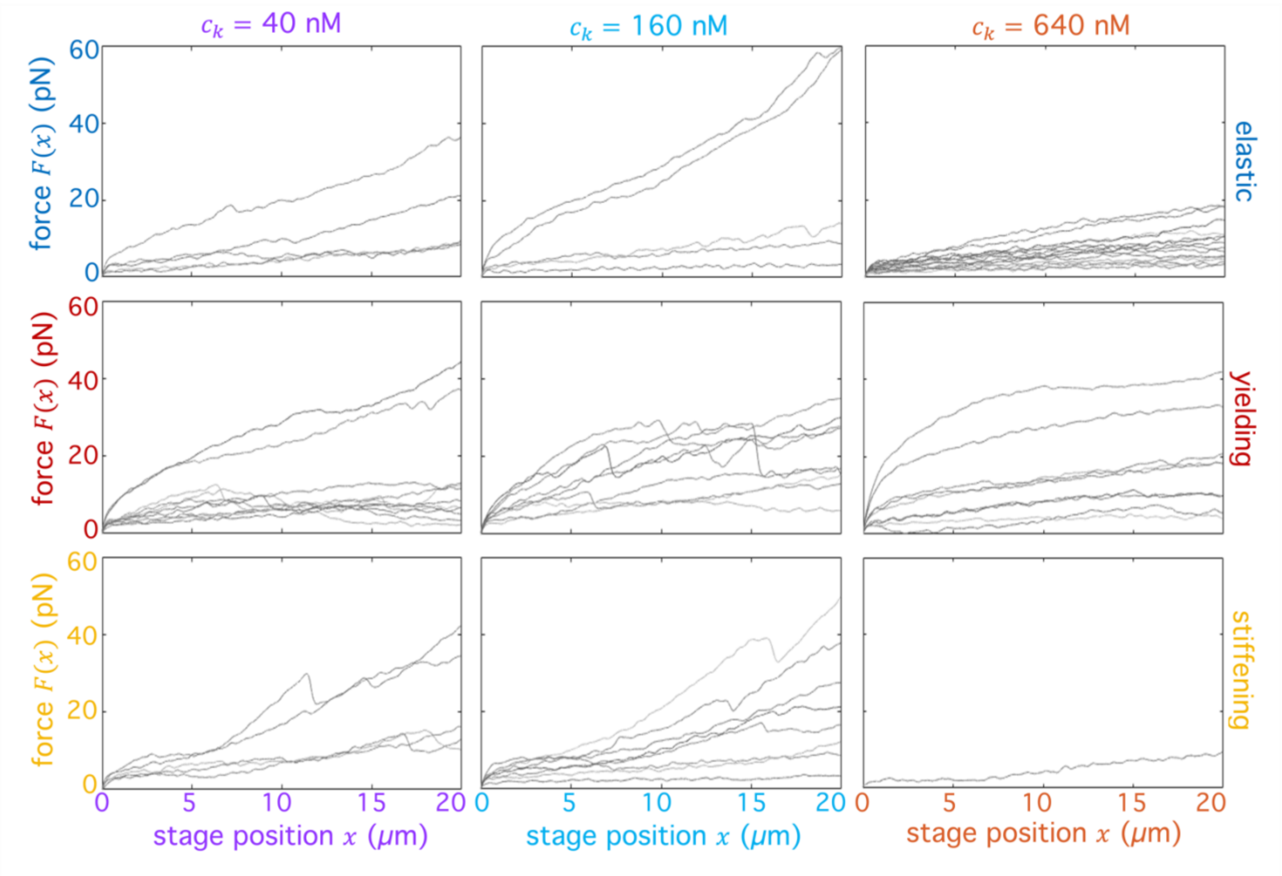

Figure S6. Ensemble of force traces comprising averages shown in Figure 4A. Individual force traces measured in response to strains with $v =$ 24 μm s^{-1} in composites with kinesin concentrations of c_k = 40 nM (left, purple labels), 160 nM (middle, cyan labels), 640 nM (right, orange labels). Traces shown compromise the average curves shown in Figure 4A for elastic (top, blue labels), yielding (middle, red), and stiffening (bottom, gold) responses at the given kinesin concentrations.

Supplementary References

1. McGorty, R. J. *et al.* Kinesin and myosin motors compete to drive rich multiphase dynamics in programmable cytoskeletal composites. *PNAS Nexus* **2**, pgad245 (2023).
2. Katira, P. https://github.com/compactmatterlab/active-filament-networks/2024.
3. Meyhöfer, E. & Howard, J. The force generated by a single kinesin molecule against an elastic load. *Proc Natl Acad Sci U S A* **92**, 574–578 (1995).
4. Vazquez-Hidalgo, E., Farris, C. M., Rowat, A. C. & Katira, P. Chemo-Mechanical Factors That Limit Cellular Force Generation. *Frontiers in Physics* **10**, (2022).
5. Howard, J. *Mechanics of Motor Proteins and the Cytoskeleton*. (Sinauer Associates, Publishers, Sunderland, Mass, 2001).
6. Robertson-Anderson, R. M. *Biopolymer Networks: Design, Dynamics and Discovery*. (IOP Publishing, 2024).
7. Gurmessa, B., Fitzpatrick, R., Falzone, T. T. & Robertson-Anderson, R. M. Entanglement Density Tunes Microscale Nonlinear Response of Entangled Actin. *Macromolecules* **49**, 3948–3955 (2016).
8. Ricketts, S. N. *et al.* Varying crosslinking motifs drive the mesoscale mechanics of actin-microtubule composites. *Sci Rep* **9**, 12831 (2019).
9. Squires, T. M. Nonlinear microrheology: bulk stresses versus direct interactions. *Langmuir* **24**, 1147–1159 (2008).
